# At the breaking point: developmental and molecular insights into *Physalis grisea* fruit abscission

**DOI:** 10.3389/fpls.2026.1733427

**Published:** 2026-02-16

**Authors:** Elise Tomaszewski, Nathan Reem, Eric Tismark Boham, Victoria Swiler, Joyce Van Eck

**Affiliations:** 1 Section of Plant Breeding and Genetics, Cornell University, Ithaca, NY, United States; 2 Boyce Thompson Institute, Ithaca, NY, United States; 3 Regeneron Pharmaceuticals, Rensselaer, NY, United States; 4 Plant and Microbial Biosciences Program, Washington University in St. Louis, St. Louis, MO, United States

**Keywords:** abscission, abscission zone, auxin, ethylene, fruit drop, groundcherry, Solanaceae, transcriptomics (RNA-seq)

## Abstract

Fruit abscission is an agronomically important trait that would benefit from a deeper molecular understanding. Despite a prominent, deleterious, fruit drop phenotype, fruit abscission has yet to be characterized in *Physalis grisea* (groundcherry). Here we established a stage-resolved timeline of *P*. *grisea* pedicel abscission zone (AZ) development to expand the general knowledge of fruit abscission. We integrated microscopic imaging of the AZ, hormone (auxin and ethylene) applications, detachment force measurements, and gene expression analysis of AZ cells across maturation to connect the role of putative regulators to cell development and separation. A strong correlation between AZ development, hormone sensitivity, and force detachment was observed. RNA-seq showed upregulation of pathways involved in cell division/expansion early in AZ development, hormone signaling and transcriptional reprogramming at the middle stage, and cell wall degradation and protective barrier genes late in the abscission process. Furthermore, MADS-box transcription factors such as the *P*. *grisea* orthologs of *JOINTLESS* and *MACROCALYX* are co-expressed during AZ differentiation, suggesting involvement in the formation of AZ cells. These results provide a molecular and cellular framework for *P*. *grisea* fruit abscission, suggesting that key regulatory features of fruit abscission are shared within the Solanaceae. Characterization of fruit abscission in *P*. *grisea* is essential for understanding this trait to guide improvements needed for its adoption as a specialty crop in the United States.

## 1 Introduction

Fruit abscission, or fruit drop, is defined as the shedding of fruit from a plant and is both an ecologically and agriculturally important process. Some plant species have evolved fruit abscission mechanisms as a means for seed dispersal (Osborne and Morgan, 1989; Estornell et al., 2013). In parallel, abscission helps to maintain an optimal carbon balance to ensure proper fruit development and ripening, which occurs in tree fruit such as citrus, apple, litchi, and macadamia (Sanz et al., 1987; Yuan and Huang, 1988; Lordan et al., 2019; Yang and Xiang, 2022).

**Figure 1:**
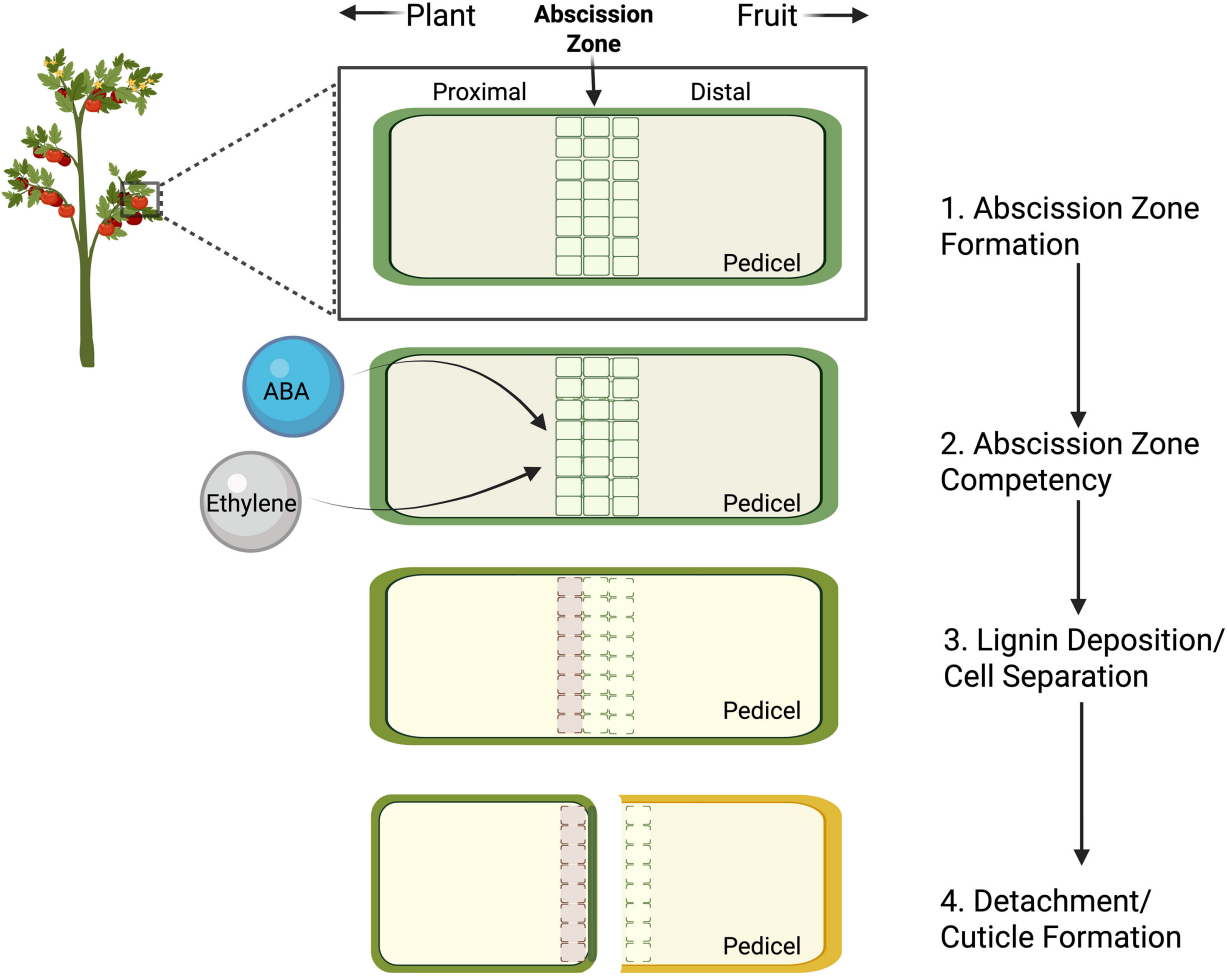
Schematic of the theorized stages of fruit abscission across development: 1) pedicel abscission zone (AZ) cell differentiation; 2) acquisition of competence to endogenous/exogenous abscission promoting signals such as ethylene or abscisic acid; 3) proximal lignin deposition and AZ cell separation via middle lamella degradation; 4) physical separation along the AZ and cuticle formation on the remaining proximal pedicel. Diagram is not to scale and steps may temporally overlap.

Among the species that exhibit fruit abscission, it generally occurs in four main stages (**Figure 1**). First, an abscission zone (AZ) develops across the organ of detachment, characterized by a region of small, cytoplasmically dense cells. Second, these cells become responsive, or competent, to endogenous and exogenous signals that trigger abscission. This is followed by lignin deposition on the proximal region of the AZ and an upregulation of cell wall degrading enzymes, resulting in dissolution of the middle lamella within the AZ. Finally, a protective cuticle barrier forms on the side of the AZ that remains upon detachment of the organ. Importantly, while these steps appear distinct, they can vary from species to species and likely overlap during abscission.

Early fruit abscission research focused on the role of plant hormones, such as ethylene and abscisic acid, which were shown to induce and accelerate abscission in different species, while auxin and gibberellic acid delayed the process (Addicott et al., 1955; Brown, 1997; Eo and Lee, 2009; Xie et al., 2013; Botton and Ruperti, 2019). It has also been shown that both biotic and abiotic stress can influence fruit abscission (Addicott, 1968). For example, low light levels, drought, and insect predation are known to induce fruit abscission (McMichael et al., 1973; Stephenson, 1981; Byers et al., 1991; Benda et al., 2009; Li et al., 2021). Not surprisingly, fruit abscission is a complex process controlled by the interplay between genetics and environmental factors.

**Figure 2:**
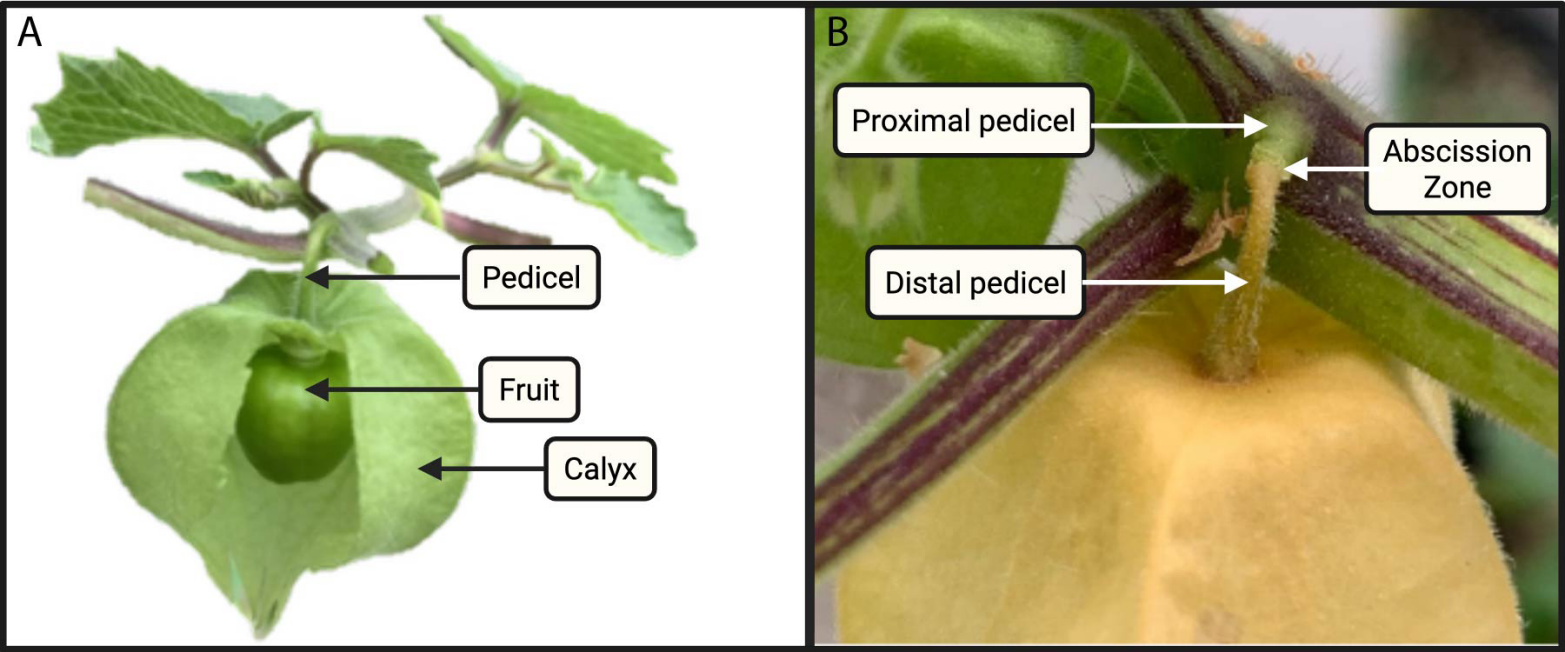
*Physalis grisea* (groundcherry) fruit and pedicel morphology. **(A)** Diagram of an unripe fruit surrounded by a calyx with attachment to the plant by a pedicel. Calyx is cut to show developing fruit inside. **(B)** Enlarged image of a ripe fruit surrounded by a calyx. The proximal pedicel remains green upon fruit maturation; the distal pedicel simultaneously senesces with the calyx. The abscission zone connects these two regions.

Fruit trees provide the most recognizable example of fruit drop, yet this phenomenon is also present in herbaceous crops such as cotton, tomato, and in an emerging non-woody model species, *Physalis grisea* (groundcherry) (Dutt, 1928; Jensen and Valdovinos, 1967; Ito and Nakano, 2015; Dale et al., 2024), which exhibits a pronounced abscission phenotype, often before the fruit is fully ripe. *P*. *grisea* fruit are distinct with an inflated calyx that surrounds the developing berry (**Figure 2**). The husk and fruit are initially green in color and then transition to yellow upon maturation. The fruit is attached to the plant via a pedicel, which also undergoes senescence upon fruit ripening. The pedicel is organized as three distinct regions: 1) proximal zone, which does not change color and remains connected to the stem upon separation, 2) abscission zone, where separation occurs and is approximately 3 mm from the pedicel/stem junction, and 3) distal pedicel, which detaches with the fruit and senesces to a yellow color.

Despite having a nutritious, edible fruit, *P. grisea* is an underutilized species native to North America, primarily grown on small-scale farms across the United States and in home gardens (Shenstone et al., 2020; Dale et al., 2024). Limited cultivation can be attributed, in part, to a sprawling, difficult to manage growth habit, but also to its persistent rate of fruit drop. This phenotype will reduce profits for growers of this crop due to the Food Safety Modernization Act (FSMA), which prohibits farmers from harvesting and selling dropped produce to reduce the risk of foodborne illness outbreaks (21 CFR Part 112 – Standards for the Growing, Harvesting, Packing, and Holding of Produce for Human Consumption, 2015). Therefore, it is necessary to have a better understanding of fruit abscission in *P. grisea* to mitigate this physiological process for increased adoption and profitability of the species on a larger scale.

Although there have been studies of abscission in several species, the underlying genetic mechanisms that control the entire process are still not fully understood. *P. grisea*, which has recently developed resources including a reference genome and plant bioengineering methods, serves as a new model species for studying fruit abscission (He et al., 2023). The research presented here leverages these resources for molecular and cellular characterization of fruit abscission in *P. grisea*. In particular, we investigated the connection between how the AZ: 1) matures on macroscopic and microscopic levels, 2) responds to the application of different hormones, and 3) undergoes transcriptional changes across development. Results from this work will broaden understanding of the molecular regulation of fruit drop, providing strategies to modulate or eliminate this trait in *P*. *grisea* and other species.

## 2 Materials and methods

### 2.1 Plant material and greenhouse conditions

*Physalis grisea* line ZL05 seeds were sown in Sunshine^®^ Mix (Sun Gro Horticulture) and maintained in growth chambers with 16 h of light (T8 fluorescent bulbs, 175 μmol), at 23 °C and 60% relative humidity until the 4-leaf stage followed by transfer to 10.2 cm (4 in) pots containing Sunshine^®^ Mix. The seedlings were transferred to a greenhouse with day and night temperatures of 26 °C and 21 °C, respectively. Supplemental lighting was provided when ambient PPFD fell below 350 µmol m^-^² s^-^¹ to maintain a 14 h photoperiod (1000 W high pressure sodium lamps). Plants were fertilized with liquid feed Peters Excel 15-5–15 Calcium-Magnesium Special (ICL) at a rate of 150 ppm 3 times weekly. After 1 month, they were transplanted into 3.79 L (1 gallon) pots with Sunshine^®^ Mix and remained under the same greenhouse parameters.

### 2.2 Fruit development time series experiment and microscopy

Five open flowers were tagged per plant with lightweight sewing thread to reduce strain on the pedicels during growth. Two plants were used per time point resulting in 10 pedicels embedded for each time point. A Nikon D3500 camera was used for photos of pedicels and fruit prior to embedding.

Paraffin embedding was modified from the protocol reported by Strable and Satterlee (2021). Modifications included pedicels that were collected into scintillation vials containing 5 mL of Formalin-Aceto-Alcohol solution (FAA) that were maintained on ice. The pedicels were vacuum infiltrated on ice for 30 min followed by a slow pressure release, and the process was repeated. Additionally, pedicel samples were infiltrated daily for 7 d in fresh Paraplast Plus (Electron Microscopy Sciences). For the embedding process, 2–3 pedicels were added per mold (Electron Microscopy Sciences Disposable Base Mold, 15 x 15 x 5 mm) and placed onto a heating block set at 60 °C. After warming for 1 min, a small amount of molten Paraplast Plus was added to the mold, covering the pedicels. Using a needle flash-warmed to 250 °C for 3 s, the pedicels were moved into the correct orientation at the bottom of the mold. The mold was carefully removed from the heating block for 15 s to start solidification. A cassette (Fisherbrand™ Histosette™ II Tissue Processing/Embedding Cassettes with Separate Lid and Base) was placed on top of the mold and additional molten Paraplast was added to seal the 2 pieces together. The full cassette was moved to ice for 5 min to rapidly solidify followed by storage at 4 °C until processed.

Paraffin blocks were trimmed using a razor blade to a parallelogram shape and mounted onto a microtome fitted with a 0.3 mm microtome blade (Fisherbrand™ Tissue Path™ High-Profile Microtome Blades) at a cutting angle of 3 degrees. Sections (10-12 μM) were made, placed into a 42 °C water bath for 5 min, and transferred to microscope slides (VWR^®^ VistaVision™ HistoBond^®^ Premium Adhesion Slides). The slides were placed on a 42 °C heating block for 15 min and transferred to a 42 °C incubator for 1 d prior to clearing the sections.

For staining with toluidine blue (Thermo Scientific Chemicals), slides were cleared and treated according to (Strable et al., 2020). Paraffin-embedded pedicel sections were mounted on microscope slides and deparaffinized by 2, 10 min incubations in clean Histo-Clear (National Diagnostics). Sections were rehydrated through a graded ethanol series: twice in 100%, 5 min; 95%, 2 min; 70%, 2 min; and 50%, 2 min; followed by a rinse in deionized water for 2 min. Slides were fixed with Permount mounting medium (Fisher Scientific) and overlain with coverslips. Slides were dried for 8 h before observation and imaged with a Leica M205 Stereomicroscope.

### 2.3 Fruit detachment force measurement

Five *Physalis grisea* plants were used for data collection. For each plant, 5 flowers per time point (0, 4, 11, 18, 25 DPA) were tagged across a 1-month period to have fruit ready for collection within the same day. Abscission force was measured using a digital force gauge fitted with a hook attachment (VTSYIQI SF-2 digital force gauge; 2 N max load [0.45 lb capacity]). Analyses were performed in R 4.3.3 (Angel Food Cake). Data were restricted to genotype ZL05 and rows with missing force values were removed. For each DPA we computed the number of observations (n), mean force (N), standard deviation, and standard error. To test whether detachment force differed across developmental time points, we first fit a one-way ANOVA with DPA as a fixed factor. Model assumptions were checked by a Shapiro–Wilk test of residuals (normality) and Levene’s test (homogeneity of variance). If assumptions were met, we used Tukey–Kramer pairwise comparisons. If assumptions were violated, inference proceeded with a Kruskal–Wallis test followed by Dunn’s *post-hoc* comparisons with Holm adjustment for multiple testing. All tests were two-sided with α = 0.05. Boxplots were created of raw forces by DPA (median, interquartile range, whiskers to 1.5×IQR; outliers as points).

### 2.4 Auxin and ethylene treatments

Six *Physalis grisea* plants were grown per treatment and randomized within the greenhouse to avoid spatial effects. Five open flowers were tagged per 2-month-old plant. After 2 weeks, ethylene or auxin applications were applied, and fruit drop was monitored daily Monday-Friday for 14 d. Each treatment was conducted in 2 experimental replicates. For auxin treatments, fruit were excised just above the husk using a sharp scalpel, leaving both the distal and proximal pedicel attached to the plant. A 1% indole-3-acetic acid (IAA; VWR, ≥98%) solution in lanolin (VWR), or lanolin alone as a control, was applied with a size 4 camel-hair paintbrush (Charles Leonard, Inc.) to either the distal or proximal pedicel. Detachment was recorded when the distal pedicel separated from the proximal pedicel. For ethylene treatments, a solution of 2000 ppm of ethephon (Qualli-Pro Ethephon 2SL) containing 2 drops of Tween-20, or a control of water plus Tween-20, was applied to 14-day old pedicels with the fruit still attached. The solution was applied with a paintbrush twice to either the distal or proximal pedicel. Detachment was recorded when the fruit separated from the plant.

Analyses were conducted in R 4.3.3. For each treatment and day after application, we calculated the percentage of pedicels remaining attached and plotted mean ± SE trajectories over time, with dotted reference lines at pre-specified evaluation days. To formally compare detachment dynamics, daily counts of fruit remaining were converted to fruit-level time-to-event data: the decrease in counts between consecutive days defined the number of detachment events assigned to that day, and fruit still attached at the final observation were right-censored. Within each hormone, Kaplan–Meier survival curves were estimated by treatment and pairwise log-rank tests were used to compare treatments; p-values were adjusted within hormone using Holm’s method for multiple comparisons. Adjusted two-sided p-values (α = 0.05) are reported in **Supplementary Table 1**.

### 2.5 RNA-seq analysis

Four bio-replicates of 3 growth stages were collected from wild-type plants grown in the greenhouse under normal, unstressed conditions. The stages were as follows:

- Early: pedicel AZ samples before flowering (no senescence).
- Mid: pedicel AZ samples after flowering (AZ senescence begins).
- Late: pedicel AZ samples prior to fruit abscission (AZ senescence complete; pedicel and husk have begun senescing).

Total RNA was isolated from the abscission zone samples using Trizol/chloroform extraction, cleaned up using an RNeasy kit (Qiagen), and 2 μg of RNA per sample was used for reverse transcription and ligation of barcode adaptors. After reverse transcriptase and barcoding, samples were sent to Genewiz for RNA-seq (one lane, paired-end reads). Read mapping and gene expression analysis were performed at Polar Genomics. Raw RNA-seq reads were processed to remove adaptors and low-quality sequences using Trimmomatic (version 0.36) with default parameters (Bolger et al., 2014). Cleaned reads shorter than 40 bp were discarded. The remaining cleaned reads were aligned to the ribosomal RNA database (Quast et al., 2013) using bowtie (version 1.1.2) (Langmead, 2010) allowing up to 3 mismatches, and those aligned were discarded. The final high-quality cleaned reads were aligned to the reference genome using HISAT2 (version 2.1.0) (Kim et al., 2019). Based on the alignments, raw read counts for each gene were calculated and normalized to fragments per kilobase of exon model per million mapped fragments (FPKM).

All downstream analyses were run in R 4.3.3 (macOS). Key packages/versions used in the below analyses include: DESeq2 1.42.1, ggplot2 3.5.2, pheatmap 1.0.13, EnhancedVolcano 1.20.0, ggvenn 0.1.10, eulerr 7.0.2, maSigPro 1.74.0, clusterProfiler 4.10.1, enrichplot 1.22.0, GO.db 3.18.0, AnnotationDbi 1.64.1, DOSE 3.28.2, fgsea 1.28.0, kohonen 3.0.12, dplyr 1.1.4, readr 2.1.5, readxl 1.4.5, patchwork 1.2.0, ragg 1.3.2.

Principal component analysis (PCA) was performed on the normalized expression values generated by the FPKM. Genes with zero FPKM across all samples were excluded. PCA was computed with prcomp in R on log2(FPKM + 1) values after centering and scaling, and the first two components were plotted with ggplot2 (Wickham, 2016). Samples were annotated by developmental stage (early, mid, late). To summarize replicate similarity, raw counts were transformed with DESeq2’s variance-stabilizing transformation, VST (Love et al., 2014), and pairwise Pearson correlation coefficients (r) were computed among all samples. Correlation matrices were visualized as heatmaps with pheatmap (Kolde, 2025), with rows and columns ordered by hierarchical clustering of 1–r. For each pairwise contrast, volcano plots were generated with EnhancedVolcano (Blighe et al., 2025) using log2 fold change on the x-axis and –log10(adjusted p) on the y-axis. Points meeting padj < 0.05 and |log2FC| ≥ 1 were highlighted to show significant genes (as described for **Supplementary Figure 3**).

Differential expression was performed with DESeq2 using the raw gene-level counts (design: stage with contrasts early vs mid, early vs late, and mid vs late) using DESeq2. Genes with very low counts were filtered prior to testing. Significance was defined as Benjamini–Hochberg adjusted p < 0.05 and |log2FC| ≥ 1, matching thresholds reported in the Results. Binary DEG sets (significant “any”, “up”, and “down” per contrast) were constructed from DESeq2 results. Overlaps across the three contrasts were visualized with Venn diagrams using ggvenn (Yan, 2025).

To identify stage-enriched gene sets, DEGs were first analyzed and classified based on consistent directionality in the two pairwise contrasts that define a peak (or trough) at that stage. Within each stage-enriched set, genes were ranked using a combined significance score calculated as the sum of –log10(adjusted p-value [FDR]) from the two defining contrasts (e.g., early vs mid + early vs late for early-enriched genes). The top 10 upregulated and downregulated genes and their putative functional descriptions were compiled per stage. Representative genes highlighted in the Results were selected from these top-ranked lists to illustrate the dominant stage-specific functional themes observed in the enrichment analyses. Expression patterns of the selected genes were plotted as a heatmap with stage annotations and grouped by assigned peak expression (**Supplementary Figure 5**).

**Figure 3:**
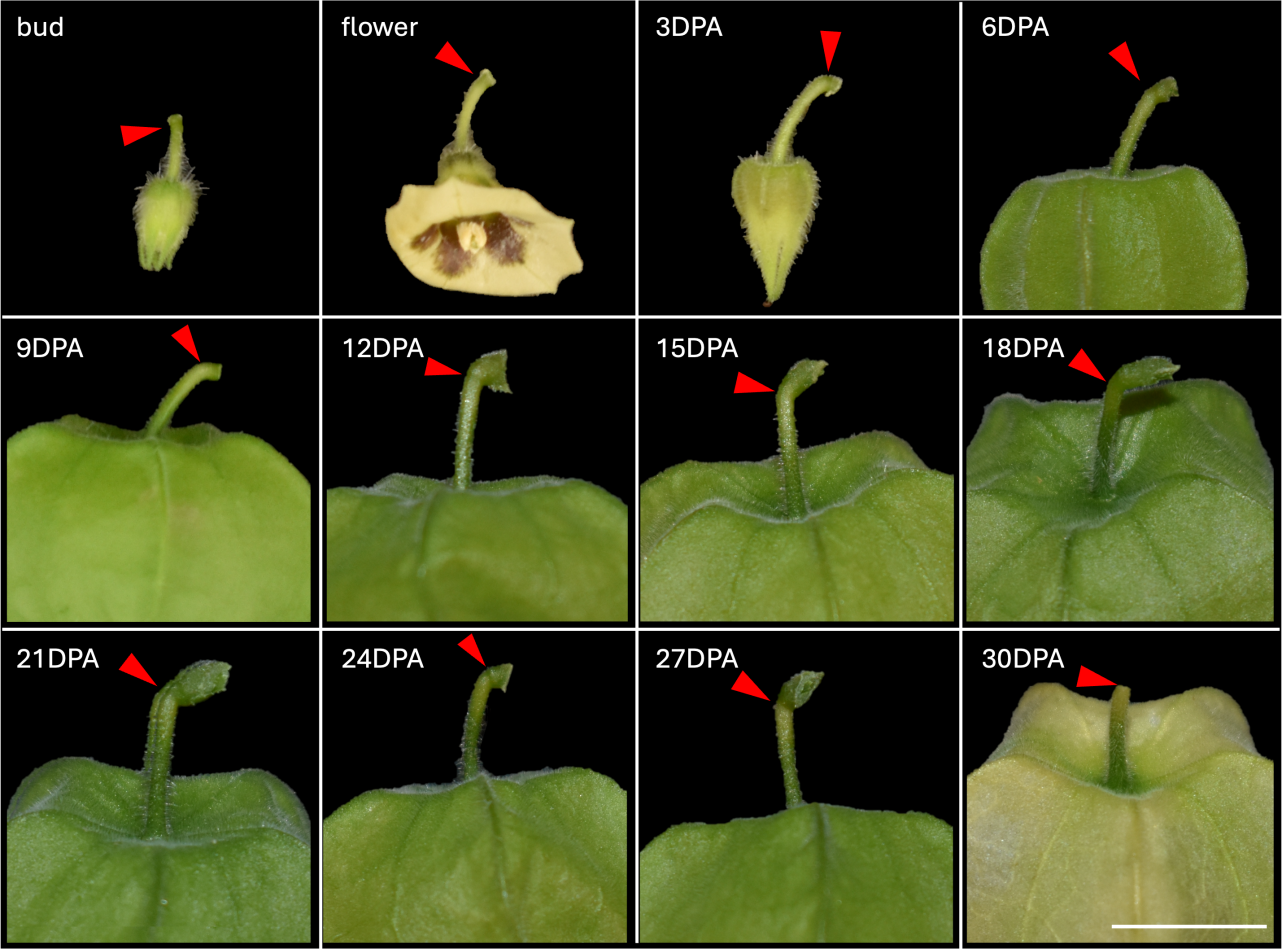
Progression of *Physalis grisea* pedicel and calyx development from bud to fruit drop at 30 days post anthesis (DPA). Red carrots indicate the location of the pedicel abscission zone. All images are scaled to the same magnification where the scale bar is equal to 1 cm.

Functional enrichment was assessed per contrast. For over-representation analysis (ORA), the universe comprised all tested genes with GO annotations defined using a custom *P. grisea* TERM2GENE mapping file; significant up- and down-regulated sets were analyzed with clusterProfiler::enricher (Yu et al., 2012; Wu et al., 2021) using Benjamini–Hochberg FDR control. For gene set enrichment analysis (GSEA), all genes were pre-ranked by the DESeq2 Wald statistic (sign-consistent with the contrast) and analyzed with fgsea (Korotkevich et al., 2016) (default permutation settings), reporting normalized enrichment scores where positive values indicate enrichment in the first stage of the contrast (as shown in **Figure 3**).

To capture stage-dependent co-expression patterns, the top 2,000 most variable DEGs (based on VST counts) were scaled per gene (row z-scores) and clustered with maSigPro (Conesa et al., 2006) into K = 8 expression profiles. The resulting gene-by-sample matrix (VST, row-scaled) was plotted as a clustered heatmap with stage and cluster annotations. As GO term coverage was incomplete for every gene in a cluster, functional enrichment was also assessed using assigned gene descriptions. Gene descriptions were derived from the closest matching tomato ortholog via nucleotide BLAST (SolGenomics ITAG 2.40), providing functional annotation for all genes in each cluster (**Supplementary Data Sheet 3**).

The 86 P. *grisea* MADS-box transcription factors were extracted from the expression matrix, averaged by stage, log-transformed as needed, and row-scaled (z-scores). A self-organizing map was trained with kohonen (Wehrens and Buydens, 2007) on these gene profiles using a 3×4 hexagonal grid, and node-level average expression curves were plotted across early, mid, and late stages. Gene-gene similarity to *PgJ* was further summarized with Pearson’s r calculated on the three-point stage-means; genes with r ≥ 0.90 were flagged as highly correlated and the corresponding tomato ortholog was based on the lowest e-value score via nucleotide sequence BLAST using SolGenomics Tomato Genome cDNA (ITAG release 2.40) as the reference.

## 3 Results

### 3.1 *Physalis grisea* fruit and abscission zone development

**Figure 4:**
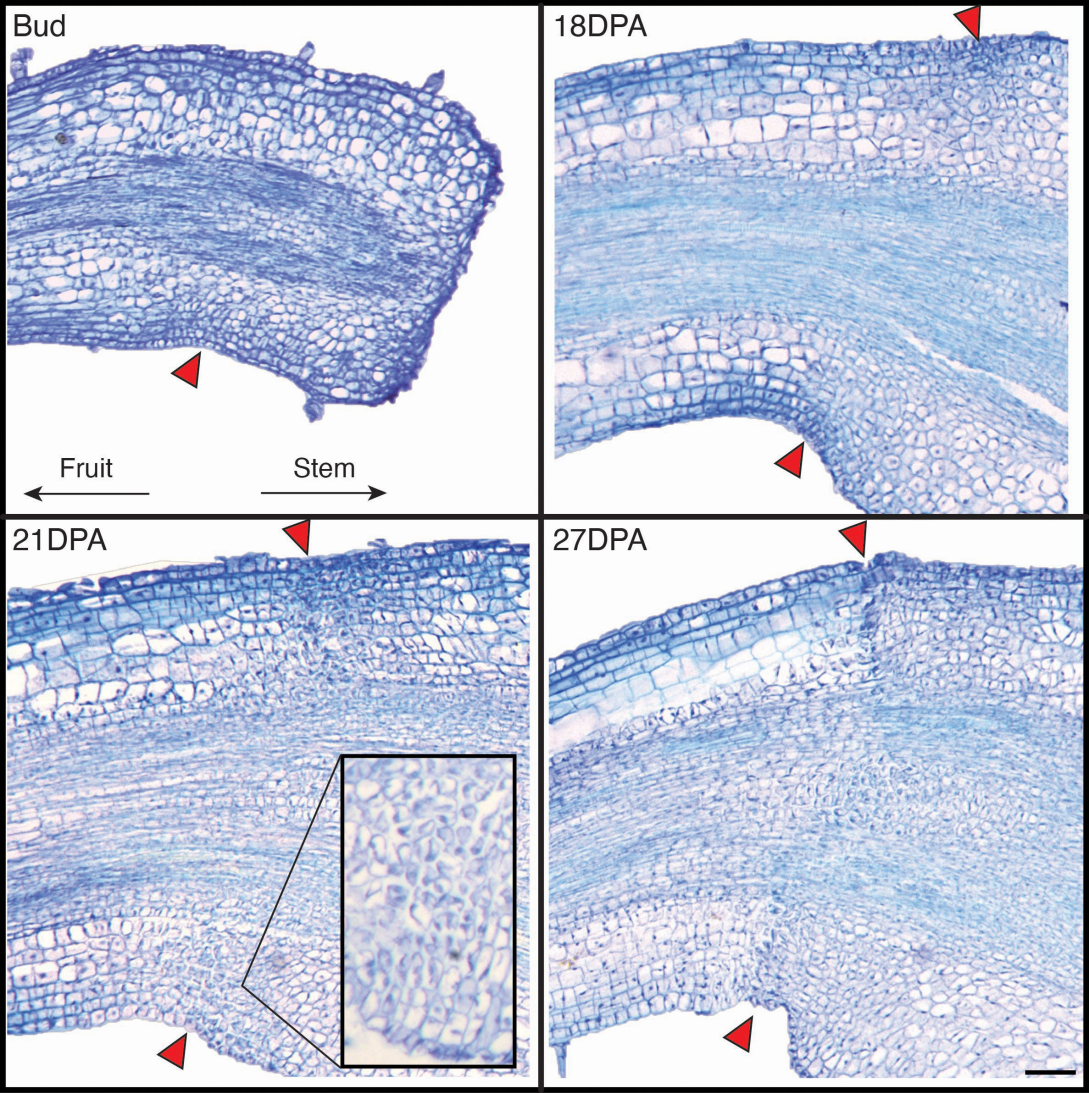
Representative images of *Physalis grisea* pedicel abscission zone cells across development (bud, 18 DPA, 21 DPA, and 27 DPA). Abscission zone cells are indicated by red arrows. All pedicels are oriented where the left side would attach to the fruit and the right side would attach to the stem. The magnified image of the abaxial pedicel in the 21 DPA panel is from the same biological sample but a different paraffin section of the larger image, illustrating intact epidermal cells and separated abscission zone cells. All images except the magnified 21 DPA are scaled to the same magnification where the scale bar is equal to 100 μm.

The earliest timepoint a fruit AZ was identifiable on *P*. *grisea* pedicels was at the bud stage, however, it was more pronounced as an indentation by 12 days post anthesis (DPA) (**Figure 4**). To correlate macroscopic changes of the pedicel AZ and husk maturation with microscopic cellular differentiation, fruit images were taken every 3 days from bud to pedicel separation. Fruit detached approximately 30 days post flowering. The calyx that surrounds the developing fruit rapidly inflated and darkened in color between 3 and 9 DPA. Although there is variation in individual pedicel thickness, it was apparent that the diameter increased until 21 DPA. At approximately 21 DPA, senescence was apparent on the distal pedicel, directly below the AZ creating a distinct differentiation between the proximal and distal regions. This senescence continued through 27 DPA, after which, the beginning of pedicel separation at the AZ was visible (image not shown). Between 27 and 30 DPA, the calyx rapidly matured to a light-yellow color, and senescence extended farther down the distal pedicel. After this maturation, the fruit detached from the plant along the AZ while the proximal pedicel remained attached to the plant.

**Figure 5:**
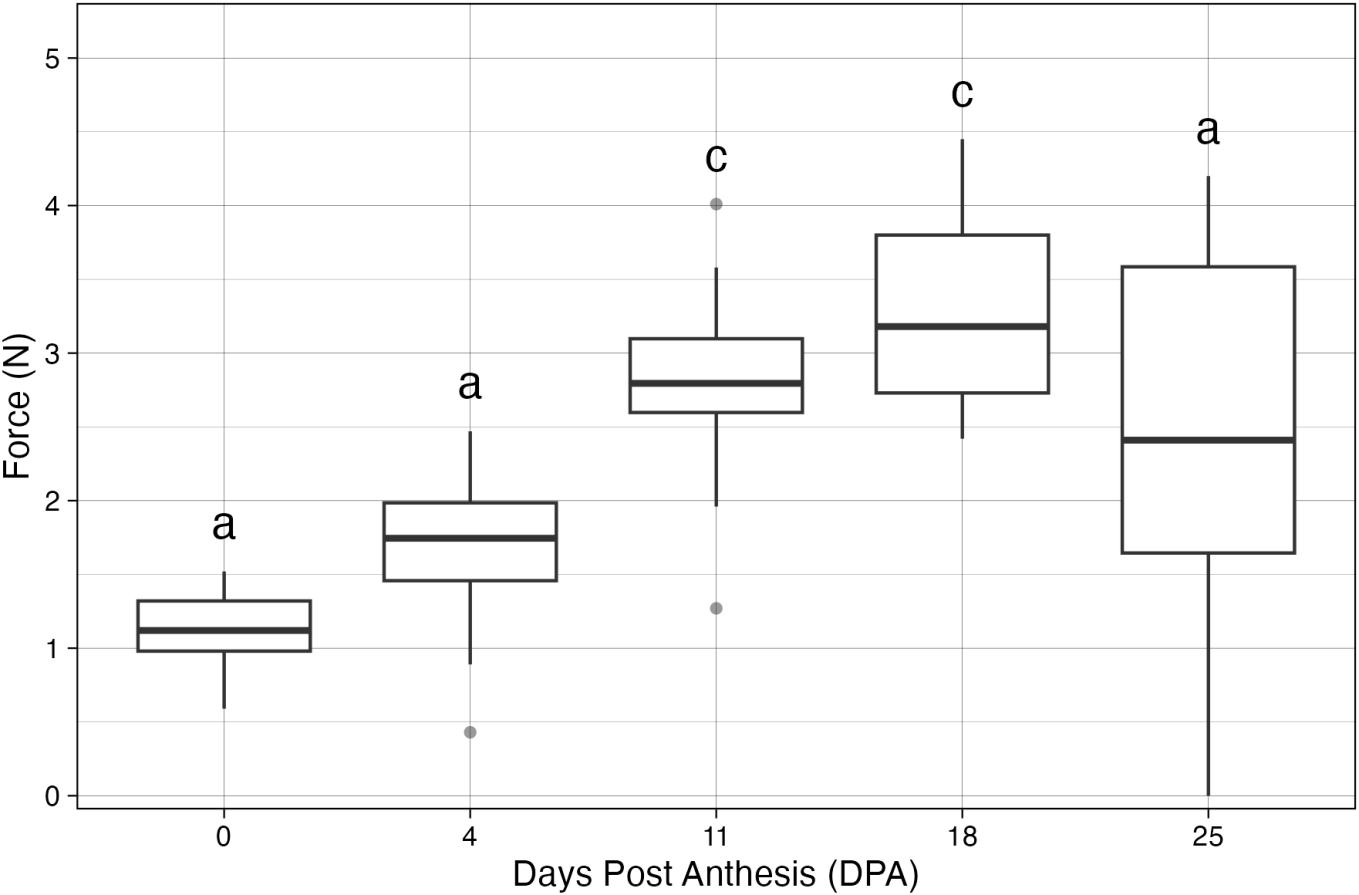
Pedicel detachment force across fruit development in *Physalis grisea*. Boxplots show the force (N) required to detach the pedicel at 0, 4, 11, 18, and 25 DPA. Sample sizes per time point: 0 DPA, *n* = 25; 4 DPA, *n* = 24; 11 DPA, *n* = 24; 18 DPA, *n* = 21; 25 DPA, *n* = 14. Groups not sharing a letter differ by Dunn’s *post-hoc* test (Holm-adjusted) following a Kruskal–Wallis test (α = 0.05).

To characterize the progression of AZ cells, microscopic images were taken of longitudinally sectioned pedicels from the stages of flower bud to abscission (**Supplementary Figure 1**). The first evidence of AZ cells was at the bud stage before anthesis, along the abaxial pedicel but not through to the adaxial side (**Figure 5**). The AZ appeared to span the entirety of the pedicel between 3 to 6 DPA (**Supplementary Figure 1**). Cell division and expansion of the AZ continued until approximately 18 DPA and ranged from 6–10 cells wide at full maturity. At approximately 21 DPA, cell separation was initiated, evident by larger intercellular spaces within the AZ. Initially, cell separation appeared more severe in the abaxial side of the AZ compared to the adaxial cells above the vasculature. AZ cells were almost completely separated by 27 DPA, creating a ridge and initiating detachment. It appeared that the AZ detached from the outer cell layers toward the inner layers. Full detachment occurred at roughly 30 DPA when the connecting vasculature separated.

### 3.2 *Physalis grisea* fruit abscission force measurements

**Figure 6:**
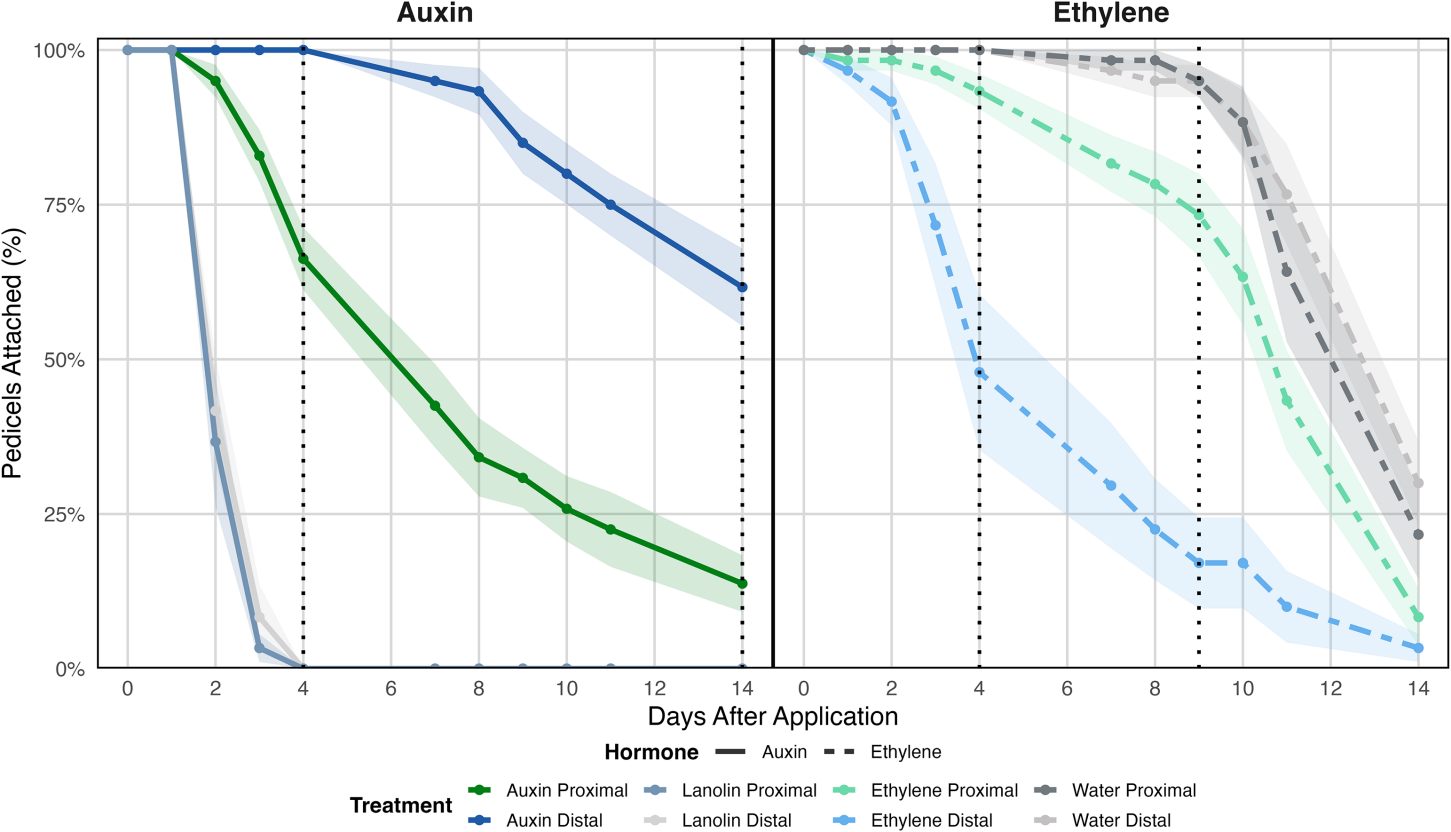
Effect of auxin and ethylene treatments on *Physalis grisea* fruit drop. Left) Mean ± SE attached pedicels after proximal vs distal 1% IAA (lanolin) application following fruit excision at 14 DPA; lanolin-only control included. Right) Mean ± SE remaining pedicels after proximal vs distal ethephon (2000 ppm + Tween-20) application with fruit attached; water + Tween-20 control included. Dotted vertical lines represent key days of comparison between the treatments and control within an application.

To detect differences in the mechanical strength of the pedicel over time, a digital force meter with a hook attachment (VTSYIQI) was used to simulate a pulling force similar to what would be required for fruit harvesting. The hook was placed around the pedicel toward the calyx, and fruit were detached across 5 developmental stages ranging from flowering to 25 DPA. The peak abscission force (Newtons, N) was recorded for each fruit. The force necessary to detach a fruit across development ranged from a mean minimum of 1.12 N at 0 DPA to a mean maximum of 3.25 N at 18 DPA (**Figure 6**). The general pattern of abscission force increased from flowering until approximately 18 DPA, and sharply decreased until fruit drop, which would naturally occur at approximately 30 DPA. The final sampling date had the greatest variance, likely due to the slight range in detachment time post anthesis causing early fruit drop and therefore less available fruit to be measured.

### 3.3 Applications of auxin and ethylene

To determine if auxin and ethylene have comparable effects on *P*. *grisea* fruit abscission as in other species, exogenous applications of both hormones were applied separately to each fruit. It is known that endogenous auxin is transported basipetally from fruit through the pedicel inhibiting abscission (Else et al., 2004; Zhao et al., 2024). Disruption in this polar auxin flow, either through a decrease in carbohydrates or ethylene induced repression of auxin flow, induces abscission (Kühn et al., 2016). Therefore, to confirm that auxin acts in a similar manner in *P*. *grisea*, 14 DPA fruit were excised just above the fruit/pedicel junction to remove the endogenous auxin source, inducing an abscission response. Auxin treatments were applied to either the distal or proximal side of each pedicel to determine if the induced abscission response could be delayed or halted. Conversely, because the fruit were not producing enough endogenous ethylene by 14 DPA to induce abscission, they were not detached for the ethylene treatments.

**Figure 7:**
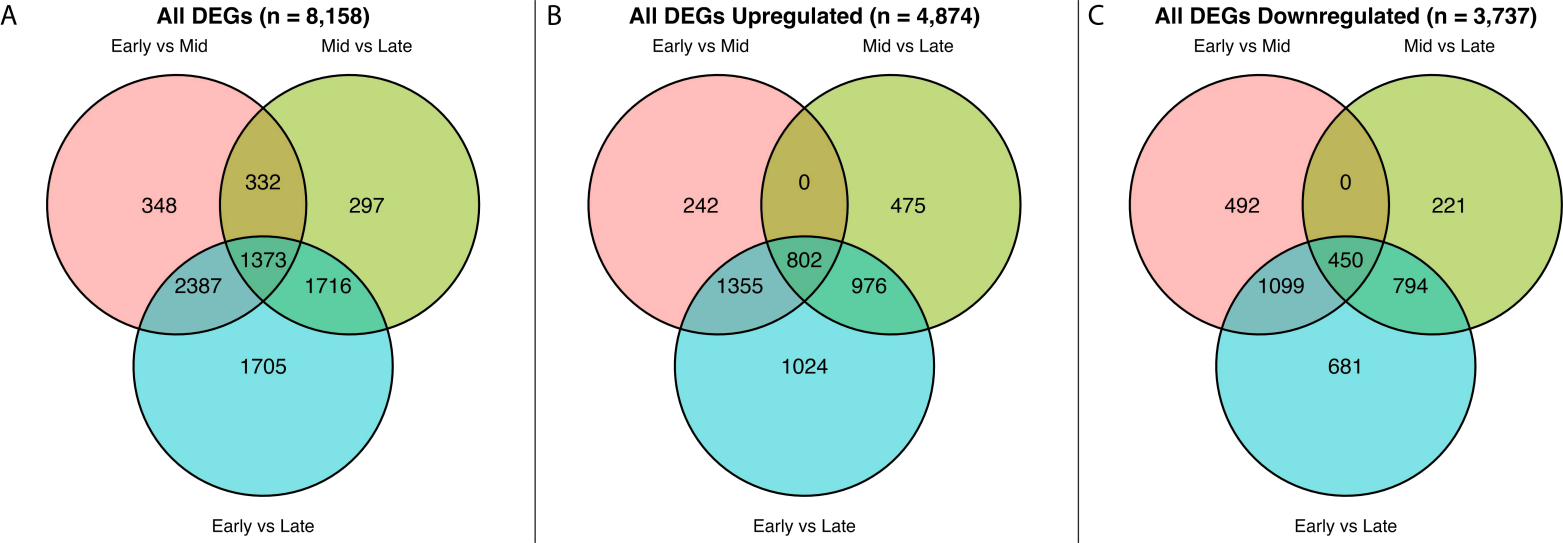
Venn diagrams of all differentially expressed genes (DEGs) from the RNA-seq data set across comparisons between the three sampling times (early, mid, and late). **(A)** All DEGs significant in at least one comparison regardless of direction. **(B)** Only upregulated DEGs significant in at least one comparison. **(C)** Only downregulated DEGs significant in at least one comparison.

Pedicels treated both proximally and distally with auxin remained on the plants longer than untreated controls; however, abscission was further delayed when it was applied to the distal side (**Figure 7**). The pedicels of controls that had fruit detached all abscised by 4 days after treatment. However, for the proximal and distal auxin treatments, 12.5% and 62% remained attached 14 days post fruit excision, respectively. In contrast, there was an inverse response when ethylene was applied to pedicels. Distal application increased the rate of pedicel separation compared to proximal application. Four days post application, 50% of the distally treated and approximately 5% of the proximally treated pedicels detached while the controls had not abscised. This trend continued until 9 days post treatment when the first control pedicels detached while 80% and 20% of the distally and proximally treated pedicels separated, respectively. Combined, these results indicate that auxin inhibits fruit abscission while ethylene accelerates it in *P*. *grisea*, similar to other species.

### 3.4 Abscission zone differential gene expression analysis across development

#### 3.4.1 RNA-seq data experimental design and quality control

To identify key genes and processes correlated with AZ development, pedicel tissue, 3 mm above and below the AZ, was collected at 3 time points: 1) early stage corresponds to bud-0 DPA pedicels that were beginning development and preceding flowering; 2) middle (mid) from approximately 15–20 DPA pedicels that were full size but with slight yellowing of the pedicel, 3) late stage from 25–30 DPA pedicels that were actively senescing but had not detached (**Supplementary Figure 2**). RNA was extracted from 4 replicates per developmental stage, where each replicate represented pooled pedicels from a single plant.

A PCA plot of the 12 AZ samples was generated using the normalized fragments per kilobases of transcript per 1 million mapped reads (FPKM) (**Supplementary Figure 3A**). The PCA plot shows clustering of the 3 different time points and their 4 replicates. Principal Component 1 (PC1) and Principal Component 2 (PC2) account for 79.7% and 12.5% respectively of the total variance. The 4 late time point replicates showed greater variability compared to the early and middle samples, with one replicate separate from the others along PC2. However, the sample-sample Pearson correlation of variance-stabilized counts showed there was high correlation between samples within the same stage (**Supplementary Figure 3B**). Library complexity and depth were uniform with a range of 10-21M reads across the 12 samples and 25.5-26.8k detected genes. Within-stage mean r was 0.968-0.979 representing high correlations between samples within a stage. No sample had an uncharacteristically low global similarity, with the lowest being a z-score of -1.61, indicating that all samples could be included for downstream analysis. Full sample quality control metrics can be found in **Supplementary Table 2**. Volcano plots were generated for each of the 3 temporal comparisons. Genes that are significant, both in log2 fold change (LFC) and p-value, are indicated by red dots on the graph (**Supplementary Figure 4**). Distribution of the significant up and downregulated differentially expressed genes (DEGs) was balanced and there was a broad range of effect sizes within each of the 3 comparisons.

#### 3.4.2 Differentially expressed gene screening for abscission zone development

**Figure 8:**
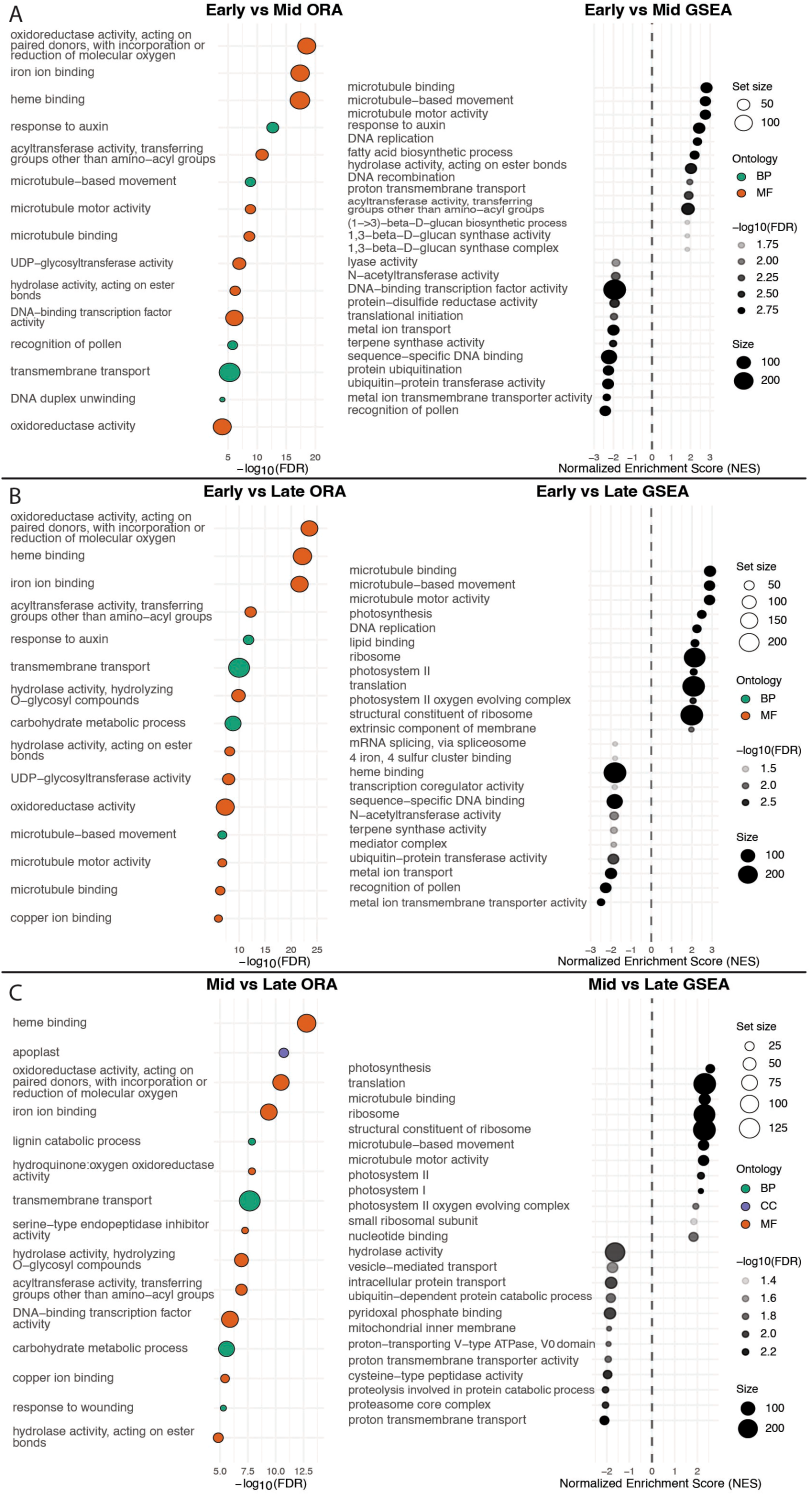
Over-representation analysis (ORA) and Gene Set Enrichment Analysis (GSEA) of the entire data set for each of the three stage comparisons. **(A)** ORA and GSEA of early vs mid expression data. **(B)** ORA and GSEA of early vs late expression data. **(C)** ORA and GSEA of mid vs late expression data. Circle size corresponds to the number of genes up or downregulated within that GO term. Point colors of the ORA graphs correspond to the ontological category of the GO term. Shading of the GSEA points represents the -log10(FDR) of that GO term. Positive Normalized Enrichment Scores correspond to the process being more upregulated in the first time point of the respective graph and negative scores correspond to processes more upregulated in the second time point.

Comparisons of transcriptomic data between the 3 time points (early vs mid, early vs late, and mid vs late) were conducted to identify DEGs enriched for a particular time point in AZ development. In total, there were 8,158 unique DEGs (padj < 0.05 & |LFC| ≥ 1) across the 3 temporal comparisons (**Figure 8**). The greatest number of DEGs was found in the comparison of early with late AZ. Whereas, the smallest number of significant DEGs was found in comparison of mid with late AZ. In total, 4,874 DEGs were upregulated and 3,737 DEGs were downregulated in at least one comparison. Notably, some genes changed direction across comparisons; therefore, the addition of all up and downregulated DEGs does not equal the total unique DEG count.

The following counts represent total DEGs per pairwise comparison, including genes that may be significant in other comparisons. In the comparison of early with mid RNA-seq data, there were 4,440 DEGs, 2,399 of which were significantly upregulated (higher in the early stage) and 2,041 that were downregulated (higher in the middle stage). In the comparison of early with late RNA-seq data, there were 7,181 DEGs, where 4,157 were upregulated (higher in the early stage) and 3,024 were downregulated (higher in the late stage). In the comparison of mid with late RNA-seq data, there were 3,718 DEGs, with 2,253 being upregulated (higher in the middle stage) and 1,465 being downregulated (higher in the late stage).

Furthermore, stage-specific genes were those with significantly higher (or lower) expression in one stage compared to both other stages. There were 3,706 early stage-specific genes (2,157 up; 1,549 down), 453 middle stage-specific genes (315 up; 138 down), and 3,022 late stage-specific genes (1,244 up; 1,778 down). The totals motivated temporal clustering and functional analysis described below. A full list of DEGs can be found in **Supplementary Data Sheet 1**.

#### 3.4.3 Stage-specific gene set enrichment analysis across early, mid, and late abscission zone development

**Figure 9:**
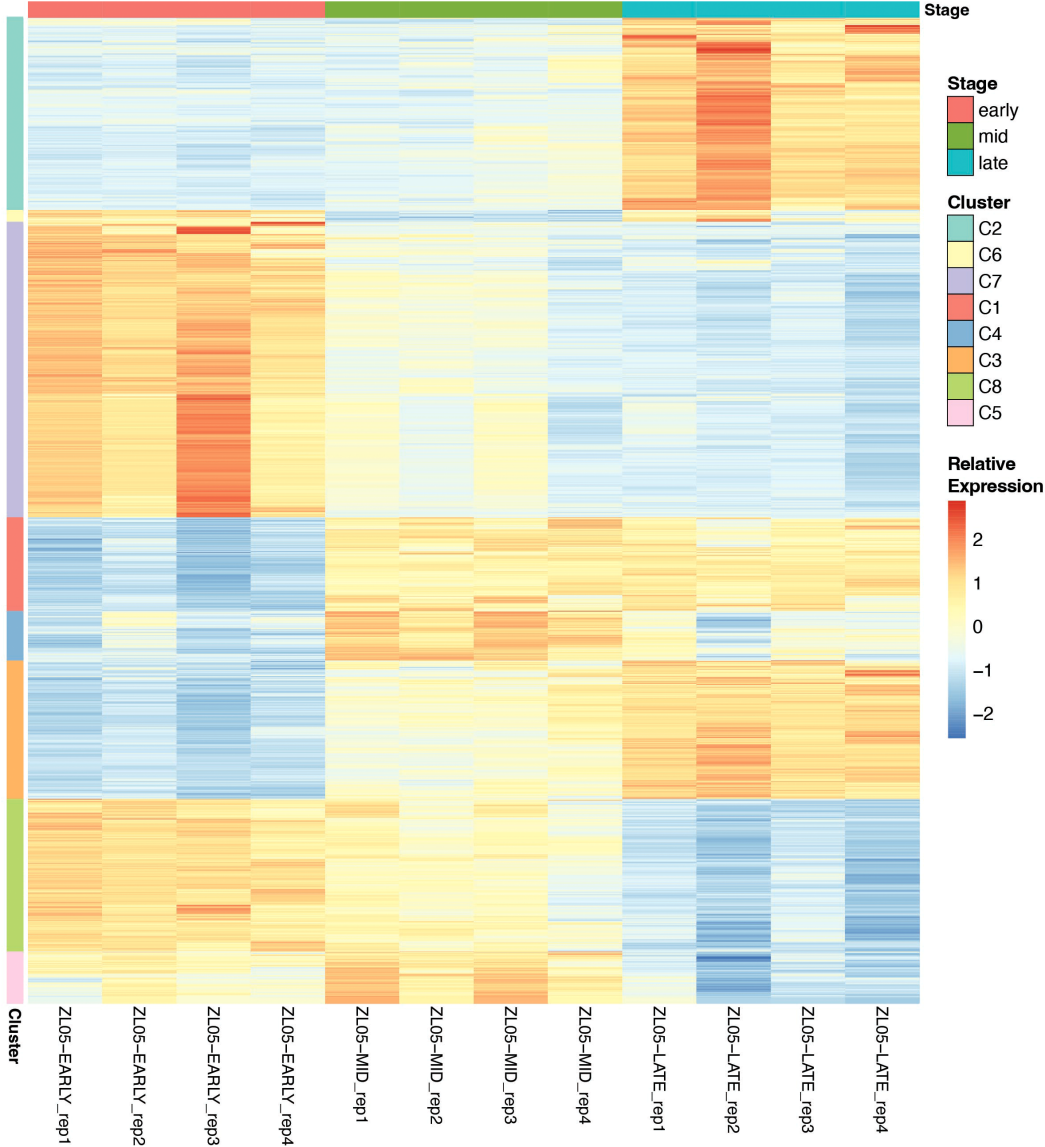
Clustered heatmap of the top 2,000 variable maSigPro-significant genes (VST, row z-score) across early, mid, and late samples. Rows were clustered into eight expression patterns (Pearson/average linkage; cutree K = 8). Top colors represent the sample stage, side colors represent the cluster, and the heat scale bar represents relative expression within each gene where blue is below the mean, white is the mean, and red is above the mean.

We first used Gene Set Enrichment Analysis (GSEA) and over-representation analysis (ORA) on stage-wise contrasts (early, mid, late) to identify global biases in GO term enrichment across the time course. Early-enriched genes were associated with cell-cycle, DNA replication, cytoskeleton and mitosis machinery, response to auxin, and biomolecule synthesis, indicating that pedicel cells are dividing and differentiating (**Figures 9A, B**). Mid-enriched genes indicated high photosynthesis, protein synthesis, and membrane transport, consistent with a metabolically active transitional phase (**Figures 9A, C**). Late-enriched sets were high in protein breakdown and turnover, oxidative processes, and cell-wall modification, indicative of AZ maturation and separation (**Figures 9B, C**).

To illustrate these patterns, representative genes from each stage are provided in **Supplementary Figure 5**. The early stage was marked by growth and hormone/transport-linked wall remodeling (kinesin-like Phygri02g013070; auxin-induced Phygri03g002780; NRT1/PTR transporter Phygri08g022580; glycine-rich wall protein Phygri04g000870; Kunitz inhibitor Phygri07g008600). The mid stage highlighted genes are associated with energy/redox metabolism and membrane trafficking/solute transport (succinate dehydrogenase Phygri01g018420; 4Fe-4S protein Phygri06g028110; cytochrome P450 Phygri10g013110; Exocyst Exo70 Phygri06g003700; SWEET sugar transporter Phygri06g006970; S-type anion channel Phygri03g031370). The late stage contained cell-wall remodeling, oxidative chemistry, and protease control genes (β-D-xylosidase Phygri11g009150; peroxidases Phygri10g011960, Phygri09g010410; glutathione S-transferase Phygri07g005250; serine protease inhibitor Phygri07g009090; UDP-glycosyltransferase Phygri08g033980; BRI1-kinase inhibitor-like Phygri08g043210; cytokinin LOG Phygri11g002310; NAC TF Phygri12g009370; C2H2 TF Phygri08g012440). These examples capture the most biologically dominant, stage-specific themes; however, full gene lists and GO terms are provided in **Supplementary Data Sheet 1**.

#### 3.4.4 Co-expressed temporal gene expression modules underlying abscission zone development

To capture co-expressed gene modules underlying these stage-specific patterns, we applied maSigPro time-course clustering to the top 2000 variable DEGs (K = 8) across all 12 samples, identifying 8 expression clusters (**Figure 3**). The 8 clusters identified two early-high/late-low modules (C07 and C08), two early-low/late-high modules (C02 and C03), three mid-peak modules (C01, C04, and C05), and one mid-low module (C06).

Gene ontology (GO) over-representation was conducted for biological interpretation; however, because not all genes in each cluster could be assigned GO terms using our custom *P*. *grisea* TERM2GENE mapping, and may affect enrichment conclusions, we supplemented GO enrichment analysis with functional enrichment based on assigned gene descriptions derived from tomato orthologs (**Supplementary Data Sheets 2**, **3**). This dual approach provided comprehensive functional interpretation of each cluster. Inspection of the most abundantly assigned GO terms and putative DEG descriptions resulted in distinct functions of the 8 clusters. Specifically, clusters with early-high expression (C07, C08) were strongly enriched for genes associated with auxin signaling, oxidoreductase activity, defense response, microtubule movement, and DNA replication, consistent with active pedicel growth and fruit retention. Kinesin-like proteins and chlorophyll a-b binding protein/subtilisin-like protease-like DEGs were the most common gene descriptions in C07 and C08, respectively. This suggests that the AZ is actively dividing and expanding, remains photosynthetically active, and is primed for peptide signaling and basal defense.

Mid-peak clusters (C01, C04, C05) were enriched for DNA-binding transcription factor activity, protein ubiquitination, transporter activity and wounding response. The most common DEGs in the mid-peak clusters included Cytochrome P450 proteins, SWEET sugar transporters, ethylene-responsive transcription factors, protein kinases, and proteinase inhibitors. Together, this indicates that the mid stage represents a period of dynamic regulatory reprogramming, adjustment of hormone and secondary-metabolite levels, redistribution of sugars and solutes, primed defense activation, and increased responsiveness to ethylene.

Clusters of late-high expression (C02, C03) were enriched for carbohydrate metabolic processes, transmembrane transport, proteolysis, lignin catabolic processes, oxidoreductase activity, hydrolase activity, metal ion transport, and heme binding. Cytochrome P450 proteins, receptor-like serine/threonine-protein kinases, F-box domain-containing proteins, peroxidases, polygalacturonases, UDP-glycosyltransferases, and RING-type domain-containing proteins were abundant. These results are consistent with detachment and wound protection of the pedicel through disassembly and remodeling of the cell wall and middle lamella, targeted turnover of regulatory proteins, lignin component modification, and ROS-mediated signaling and defense. Combined, these temporal clusters provide a global view of AZ development, from early proliferative growth to late wall degradation and defense, in agreement with the anatomical and force measurements described above.

#### 3.4.5 MADS-box transcription factor differentially expressed genes

MADS-box transcription factors are associated with regulation of fruit abscission. Specifically, previous reports link the MADS-box transcription factors *JOINTLESS* (*J*) and *MACROCALYX* (*MC*) to pedicel AZ differentiation in tomato and apple (Nakano et al., 2012, Nakano et al., 2015). Therefore, we focused our investigation on the expression of *P*. *grisea* MADS-box transcription factors to determine their involvement in the stages of fruit abscission. Of the 86 identified MADS-box genes in *P*. *grisea*, 22 were either significantly upregulated or downregulated in at least one pedicel developmental stage (**Supplementary Figure 6**). However, *J* or *MC* (designated as *MPF3* in *P*. *grisea*) were not included in any of these comparisons. The largest grouping of MADS-box DEGs included those significant in early vs mid and early vs late comparison overlap, meaning they are most significant in the early stage. Additional genes characterized for involvement in tomato AZ development include *GOBLET* (*GOB*), *BLIND*, *LATERAL SUPRESSOR* (*LS*), and *WUSCHEL* (Nakano et al., 2013, Nakano et al., 2014). Interestingly, only the *P*. *grisea* orthologs of *GOB* (Phygri07g001070) and *LS* (Phygri07g004640) were preferentially upregulated in the early stage.

**Figure 10:**
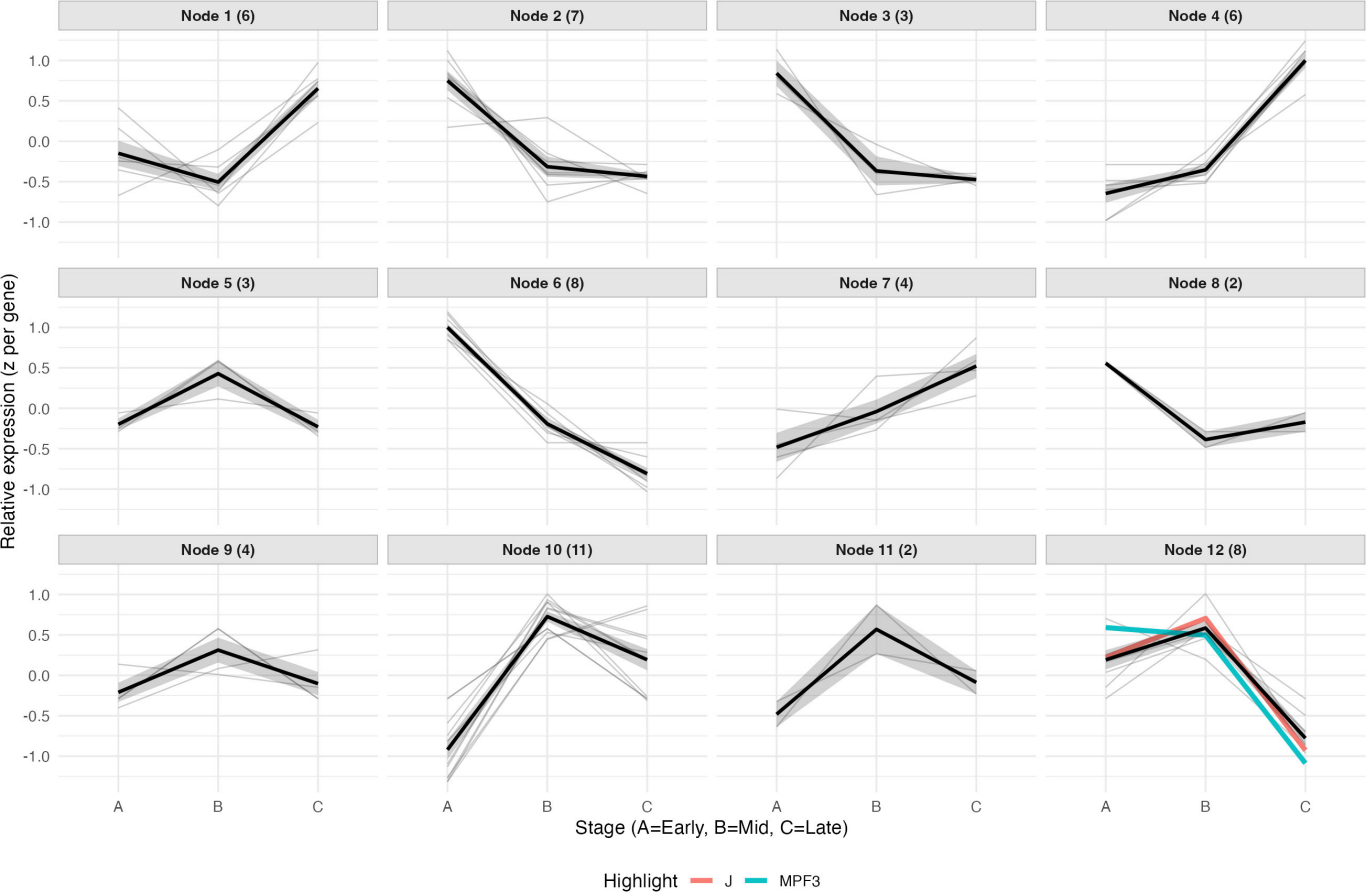
Self-organizing maps (SOMs) of the 86 identified *Physalis grisea* MADS-box transcription factors. Average expression of each gene plotted across early: A, mid: B, and late: C stages. The average expression level of each cluster (node) is denoted by a solid black line and the number of genes in each node are denoted in parentheses next to the node. Expression levels of *PgJ* and *PgMPF3* are in Node 12 and marked in red and blue, respectively.

Although not statistically significant in global temporal comparisons, expression clustering through self-organizing maps (SOMs) of the 86 P. *grisea* MADS-box genes identified that *PgJ* and *PgMPF3* clustered together (**Figure 10**; **Supplementary Data Sheet 4**). Both genes were represented in the node with the average expression pattern of high early expression, a peak in the mid stage, and low, late expression. Additional genes in this cluster included: Phygri02g025310 (*SlMBP10*), Phygri04g004530 (*SlMADS23*), Phygri07g009060 (*TM8/TDR8*), Phygri07g015380 (*SlMADS98/SlCMB1*), Phygri08g005970 (*SlMADS51*), Phygri12g018340 (*RIN*).

Another MADS-box transcription factor known for its role in tomato AZ differentiation is SlMBP2*1* (J-2), which interacts with J and MC through a heterodimer protein complex to specify the pedicel AZ (Liu et al., 2014). Notably, using nucleotide and amino acid sequence alignments of *J-2* to the *P*. *grisea* genome did not detect a clear one-to-one *J-2* ortholog. Although nucleotide and amino acid sequence alignments alone cannot definitively rule out the presence of a highly diverged ortholog, these results suggest that this ortholog is not present in *P*. *grisea* or that any related paralog has diverged substantially. Therefore, we explored other potential co-regulators with *PgJ* and *PgMPF3* within our dataset. Based on expression correlation analysis of the 86 identified MADS-box genes in *P*. *grisea*, there were several genes with high correlation to *PgJ* (r > 0.9). The genes with a ranked (most to least) relatedness to *PgJ* included: Phygri08g005970 (*SlMADS51*), Phygri02g025310 (*SlMBP10*), Phygri08g018110 (*SlMBP6/SlAGL6*), Phygri07g015380 (*SlMADS98/SlCMB1*), Phygri12g018350 (*MC*), and Phygri07g009060 (*TM8/TDR8*) (**Supplementary Table 3**).

## 4 Discussion

Organ abscission has been rigorously studied for over 100 years, from early physiological and anatomical descriptions to modern models of AZ structure and detailing influences of biotic and abiotic stressors (Inman, 1848; Jensen and Valdovinos, 1967; Addicott, 1968; González-Carranza et al., 1998; Jinn et al., 2000; Roberts et al., 2000, Roberts et al., 2002). More recently, research in *Arabidopsis* and tomato has focused on the underlying genetics of floral abscission, including the IDA–HAE/HSL2 response pathway and subsequent MAP kinase cascades (Jinn et al., 2000; Butenko et al., 2003, Butenko et al., 2012; Cho et al., 2008; Stenvik et al., 2008; Patharkar and Walker, 2015, PatharkarWalker, 2018; Lu et al., 2023). Substantial progress has also been made on understanding fruit abscission, especially in tomato and a limited number of woody fruit crops, yet it still remains less characterized compared to leaf and flower abscission (Xie et al., 2013; Shi et al., 2023; Zhao et al., 2024; Tranbarger and Tadeo, 2025). Nevertheless, recent case studies in litchi, citrus, almond, macadamia, pear, mulberry, and olive reflect a shift toward studying diverse species to reveal fruit-specific regulatory components (Gil-Amado and Gomez-Jimenez, 2013; Merelo et al., 2017; Li et al., 2019; Zhang et al., 2019; Guo et al., 2021; Haim et al., 2021; Yang and Xiang, 2022; Zheng et al., 2024; Yang et al., 2025). Collectively, these studies illustrate that fruit abscission regulation differs from flowers and leaves demonstrating that key genes and signaling networks can diverge across organs and species, including between monocots and dicots (Roberts et al., 2000; Estornell et al., 2013; Patharkar and Walker, 2018; Li and Su, 2024). Therefore, expanding fruit abscission studies in non-model and underutilized species is essential for discovering novel regulatory pathways that could modulate or eliminate fruit drop in diverse crops to broaden our food supply. Here, we address this gap by focusing on *P*. *grisea*, an underutilized fruit crop. We integrated cellular, mechanical, hormonal, and transcriptomic approaches to define a developmental timeline for the pedicel AZ. Together, our results support a stage-resolved model of *P*. *grisea* fruit abscission wherein there is early AZ specification, a mid-stage competence and reprogramming phase, and late enzymatic separation coupled with plant defense upregulation.

Reports from Tabuchi (1999) on AZ cellular development in tomato showed that there is differentiation early in pedicel development, which supports our findings as AZ cells in *P*. *grisea* are first visible near the abaxial epidermis in the bud stage. Next, AZ differentiation is present along the adaxial epidermis around 6 DPA and finally spans through the vasculature before 12 DPA. This cell division and expansion also coincide with the increase in detachment force until 18 DPA. Pedicel strengthening throughout fruit development is important to support the mass of the fruit as it grows to maturity. In addition, at approximately 18 DPA, pedicels start to senesce preceding visible middle lamella degradation and larger intercellular spaces at around 21 DPA. Whether pedicel senescence in *P. grisea* is ethylene-dependent or ripening-independent remains unclear; notably, tomato *jointless***/***jointless-2* mutants lacking a pedicel AZ still undergo pedicel senescence, implying that senescence can proceed in the absence of AZ identity (Szymkowiak and Irish, 1999; Tucker and Kim, 2015).

Our hormone treatments further confirmed the role of auxin and ethylene in regulating the transition from attachment to separation in *P*. *grisea* fruit. Consistent with models from tomato (Dong et al., 2021), we propose that sustained basipetal auxin flux maintains fruit attachment by limiting ethylene responsiveness, while a perturbation in auxin transport permits ethylene perception and initiates separation. When *P*. *grisea* fruit were removed, eliminating the endogenous auxin source and producing ethylene via wounding, the pedicels detached within a few days. However, exogenous auxin application preserved attachment and delayed abscission, demonstrating that a maintained auxin flow in the pedicel facilitates fruit retention. Conversely, with the fruit attached, exogenous ethylene accelerated fruit drop even in half-matured fruit (14 DPA). Distal applications of either hormone produced a stronger response than proximal applications, consistent with basipetal hormone transport. Together, these outcomes explain a competency window where waning auxin flow allows ethylene-driven separation to occur. More detailed time course studies are needed to define the precise timing of when this hormone transport and sensitivity transition occurs in *P*. *grisea* pedicels.

In *P*. *grisea*, early expression of genes involved in cell wall biosynthesis, cytoskeleton organization, DNA replication, and auxin signaling, is consistent with an actively dividing AZ and repression of separation. In contrast, the mid stage showed a decline in auxin signaling, a rise in ethylene response factors, membrane-trafficking processes, and transcription factor activity, aligning with metabolic reprogramming and ethylene competence without detachment. Work in tomato has indicated that lignin deposition on the proximal AZ precedes the induction ethylene-responsive cell wall-degrading enzymes, thus creating a clean position of degradation for detachment (Lee et al., 2018). In our dataset, our mid stage clusters were not enriched for lignin-specific genes, whereas our late stage modules captured lignin remodeling/breakdown, indicating that active lignin biosynthesis gene expression may have peaked outside of our sampled time points, so we cannot exclude an analogous lignin deposition phase as described in tomato. Additionally, our results show that the late stage had an upregulation of hydrolases, oxidative pathways, protein transport, and cuticle/defense programs, aligning with active degradation of the middle lamella, tissue separation, and wound protection. Together, our macroscopic observations, histology, and hormone treatments align with our RNA-seq based model of an early growth pattern, a middle reprogramming and competency establishment stage, and a late degradation and protection phase in the *P*. *grisea* pedicel AZ.

Our transcriptomic data also revealed temporal patterning of the AZ is broadly consistent with abscission programs reported in other species. Stage-specific and hormone-induced AZ transcriptomes have been described in tomato (Meir et al., 2010; Nakano et al., 2013; Wang et al., 2013; Sundaresan et al., 2015; Dong et al., 2022) and in non-tomato crops such as olive, citrus, oil palm, and others (Gil-Amado and Gomez-Jimenez, 2013; Merelo et al., 2017; Zhao et al., 2019; Fooyontphanich et al., 2021; Guo et al., 2021; Qiu et al., 2021; Sriskantharajah et al., 2021; Ma et al., 2024). Although the species differ in these studies, experimental design, transcriptomic analysis, and functional enrichment reporting, general comparisons can still be made of broad functional enrichment themes. To facilitate comparison, we examined whether functional enrichment themes from our stage-enriched DEG analysis (**Figure 9**) and our clustered co-expression analysis (**Supplementary Data Sheets 2**, **3**) appeared in these published studies. By comparing our enriched GO terms and gene descriptions to the functional enrichment themes of these studies, we identify a recurring abscission pattern of early hormone-regulated growth, a transitional phase, and late cell separation with wound protection, alongside *P. grisea*-specific features. For example, the Dong et al. (2022) study, which is the most comparable to our experimental design, indicated an enrichment for auxin signaling genes (AUX/IAA) and metabolic/oxidation-reduction processes during AZ growth, similar to the auxin- and metabolism-related themes found in our early and mid-clusters. In contrast, early *P*. *grisea* AZ development was more strongly dominated by cell-cycle and mitotic GO terms, suggesting that early AZ development in *P*. *grisea* is more overtly proliferative than in tomato.

Additionally, the auxin-depletion and ethylene-induced tomato abscission microarrays of Meir et al. (2010); Wang et al. (2013) support the mid-to-late transition and separation phase of abscission in *P. grisea* where we also see enrichment of polygalacturonases, cellulases, cell-wall hydrolases, ethylene response genes and defense related pathways. Non-tomato fruit abscission transcriptomic analyses, such as the work from Gil-Amado and Gomez-Jimenez (2013) in mature abscising olives, likewise report increased cell-wall degradation and remodeling pathways, oxidative and defense genes, and a redistribution of solutes and ions through transmembrane and metal-ion transporters. However, they found a prominent role of sterol/sphingolipid-containing membrane microdomains and ABA-associated MYB/bZIP transcription factors in regulating cell separation while *P*. *grisea* late cluster emphasized lignin catabolism, heme-binding oxidoreductases, and F-box/RING E3 ligases within an auxin–ethylene framework. These cross-species comparisons suggest that while shared abscission-associated transcriptional programs are observed across diverse plant species, especially regarding hormone-driven pedicel growth, cell separation, and wound protection, the regulation of these processes also includes species- and organ-specific components.

Due to the important role that *JOINTLESS* (*J*) and *MACROCALYX* (*MC*) play in AZ differentiation in tomato, combined with the apparent absence of a *J-2* ortholog in *P*. *grisea*, we focused on the co-expression of MADS-box transcription factors that may interact with these orthologous genes to regulate AZ positioning and differentiation. Importantly, we found that the orthologs of these two genes are co-expressed with an early-mid peak and a late stage drop, indicating their involvement with AZ identity and differentiation rather than competency acquisition or separation induction. Within the same co-expression node, *P*. *grisea* orthologs of tomato *SlMBP10*, *SlMADS23*, *TM8/TDR8*, *SlMADS98*, and *SlMADS51* were identified, appearing as potential co-regulators of AZ differentiation. Interestingly, neither *PgJ* nor *PgMPF3* was significantly differentially expressed in any of our pairwise stage comparisons, likely due to peak or critical expression occurring earlier than our first sampling point rather than a lack of involvement in AZ development. Overall, this MADS-box module appears consistent with tomato in which *J* and *MC* act upstream of cell wall degradation- and defense-enriched clusters to position and specify the AZ.

To summarize, our study has contributed to an understanding of the *P*. *grisea* fruit abscission process at stages of pedicel development. Findings showed that early in development before anthesis, the AZ cells differentiate at a predetermined location and auxin-driven separation repression is present. When cell division is complete, cell expansion and cell wall enforcement continue, strengthening the pedicel to support the maturing fruit until approximately 18 DPA. After which, endogenous auxin levels significantly decline leading to ethylene signaling competence, which upregulates cell wall degrading enzymes to initiate detachment, and separation occurs at approximately 30 DPA. Together, these findings highlight that *P. grisea* fruit abscission is a complex, highly regulated process. Although our data provides a clear understanding of regulatory mechanisms governing *P*. *grisea* fruit abscission, the limited range of sampling dates for transcriptomic results likely understate key transient regulators of the AZ. Higher resolution spatial and temporal assays could sharpen the regulatory map and provide a more detailed staging system to describe precise transitions between the abscission steps.

Leveraging genetic and transcriptomic knowledge provides opportunities through commonly used approaches and new breeding technologies to fine-tune developmental processes underlying fruit abscission in *P*. *grisea*. For example, application of ethylene is a common method to induce abscission in tree fruit (Bukovac, 1971; Edgerton, 1971; Taheri et al., 2012). Our results from applications of auxin and ethylene suggest that the same treatments could be applied to *P*. *grisea* by applying auxin to delay or ethylene to accelerate fruit drop and promote uniform ripening. Furthermore, advances in gene editing technologies allow rapid improvement for a desired ideotype by targeting specific gene families and pathways that regulate abscission. Interrupting the progression of fruit abscission prior to detachment could, in theory, reduce or eliminate fruit drop. For instance, our results suggest that modifying expression to extend basipetal auxin flow, or downregulating cell wall hydrolases or ethylene response factors could delay fruit abscission in *P*. *grisea*. However, it is important to note that potential unintended effects, such as disruptions in fruit ripening or diseased organ shedding may occur. Moreover, because many abscission-related genes belong to large families, functional redundancy and genetic compensation should be considered when designing new breeding technology strategies.

## 5 Conclusion

To our knowledge, this is the first characterization of the *Physalis grisea* pedicel AZ cellular architecture, along with integration of mechanics, hormones, and transcriptomics. Our findings support the model of a staged fruit drop in which there is early AZ differentiation with auxin-maintained attachment, a mid-stage competence and reprogramming phase, and late hydrolase-mediated degradation with cuticle biosynthesis and wound protection prior to separation. Additionally, co-expression analysis of *P*. *grisea* orthologs of *JOINTLESS* and *MACROCALYX* suggest candidate partners of early AZ differentiation. These data are consistent with shared features of fruit abscission in other previously studied crops. Results from our study inform crop improvement strategies, including timed hormone applications and genome editing targets to mitigate fruit drop both in *P*. *grisea* and other plant species. 

## Data Availability

The datasets presented in this study can be found in online repositories. The names of the repository/repositories and accession number(s) can be found below: https://www.ncbi.nlm.nih.gov/, PRJNA1347931, https://github.com/emt236/Phygri_AZ_RNAseq.
