# Supplementary material for: At the breaking point: developmental and molecular insights into Physalis grisea fruit abscission
Source: Front Plant Sci. 2026 Feb 16;17:1733427. doi: 10.3389/fpls.2026.1733427 (PMC12950737; doi:10.3389/fpls.2026.1733427)
Supplement: Supplementary file 5 [file DataSheet5.docx]

Supplementary Material

**
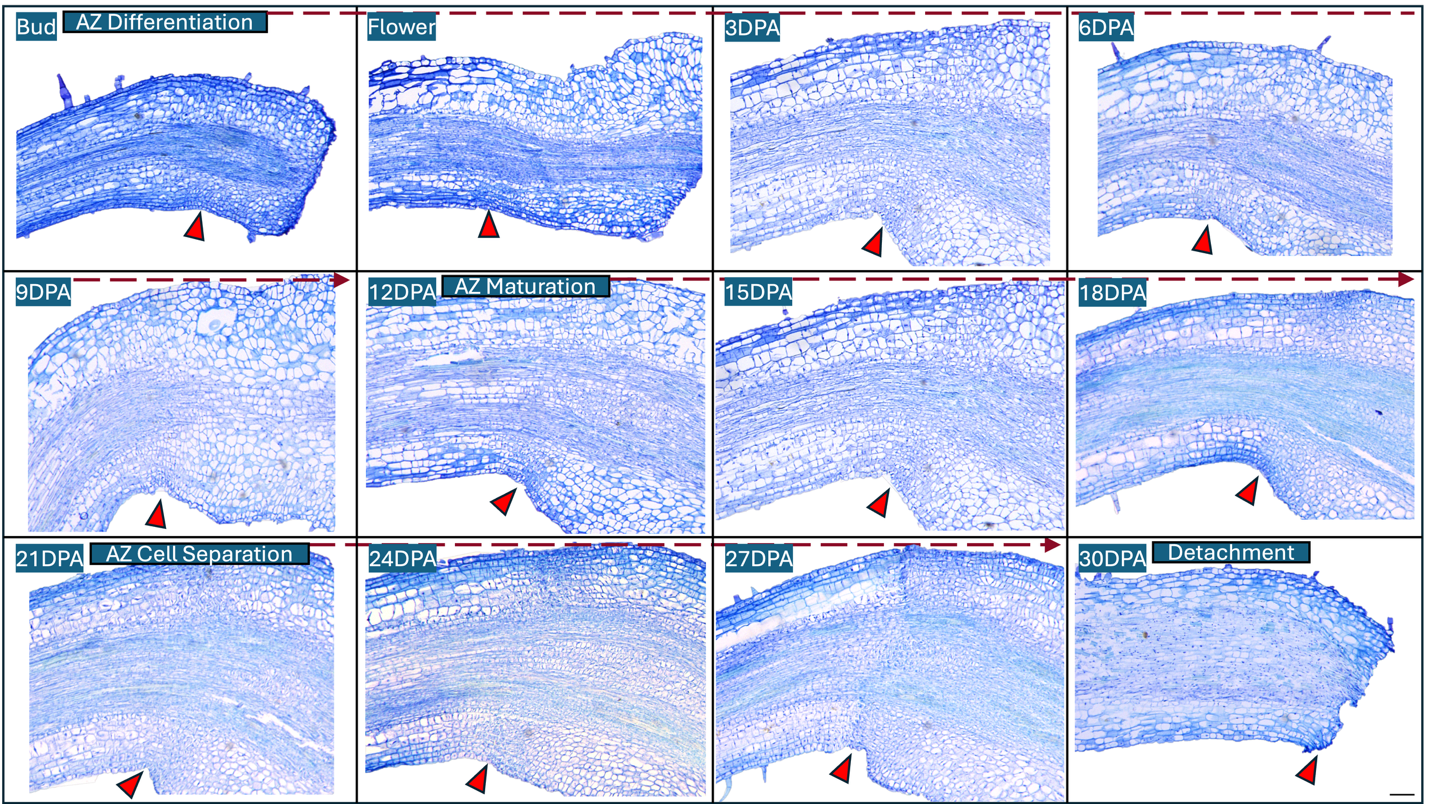
**

**Supplementary Figure 1.** Sections of *Physalis grisea* pedicels from flower bud through detachment at 30 DPA. Representative sections are 10-12 μm thick and stained with toluidine blue. Images are all scaled equally where the scale bar is equal to 100 μm. Abscission zone cells are represented by red carrots. Defining characteristics of abscission zone development are indicated by teal labels and maroon dashed arrows that span their respective time period.

**
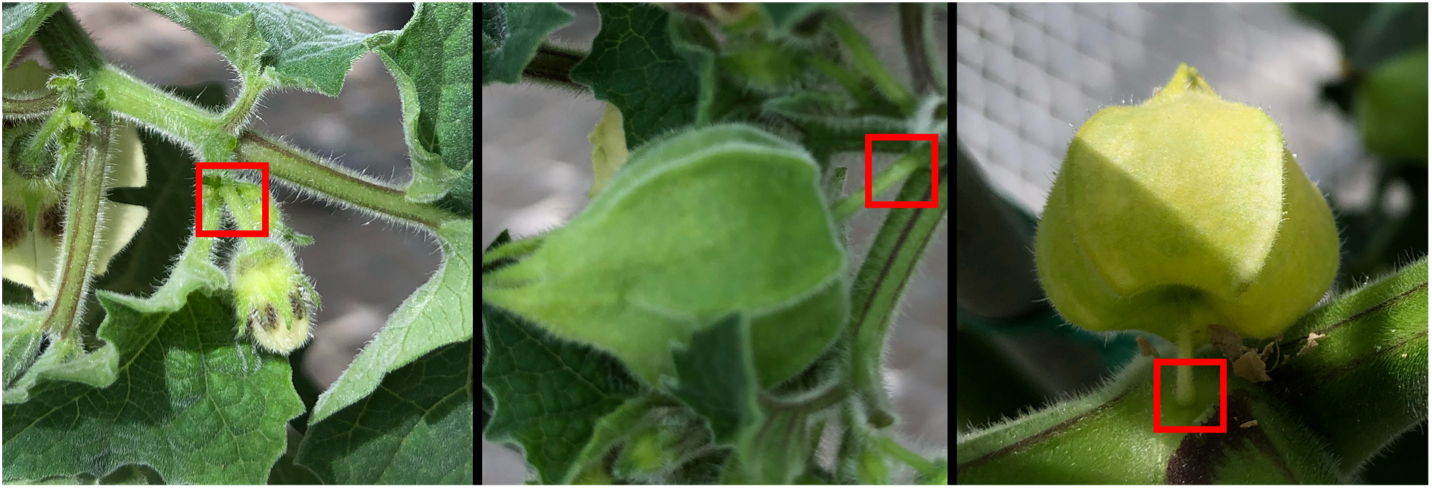
**

**Supplementary Figure 2.** Abscission zone developmental stages collected for RNA-seq analysis. **(Left)** Early stage; prior to flowering and no yellowing of the pedicel. **(Middle)** Middle stage; shortly after flowering with a green calyx and slight yellowing of the pedicel. **(Right)** Late stage; both the pedicel and calyx were yellow.


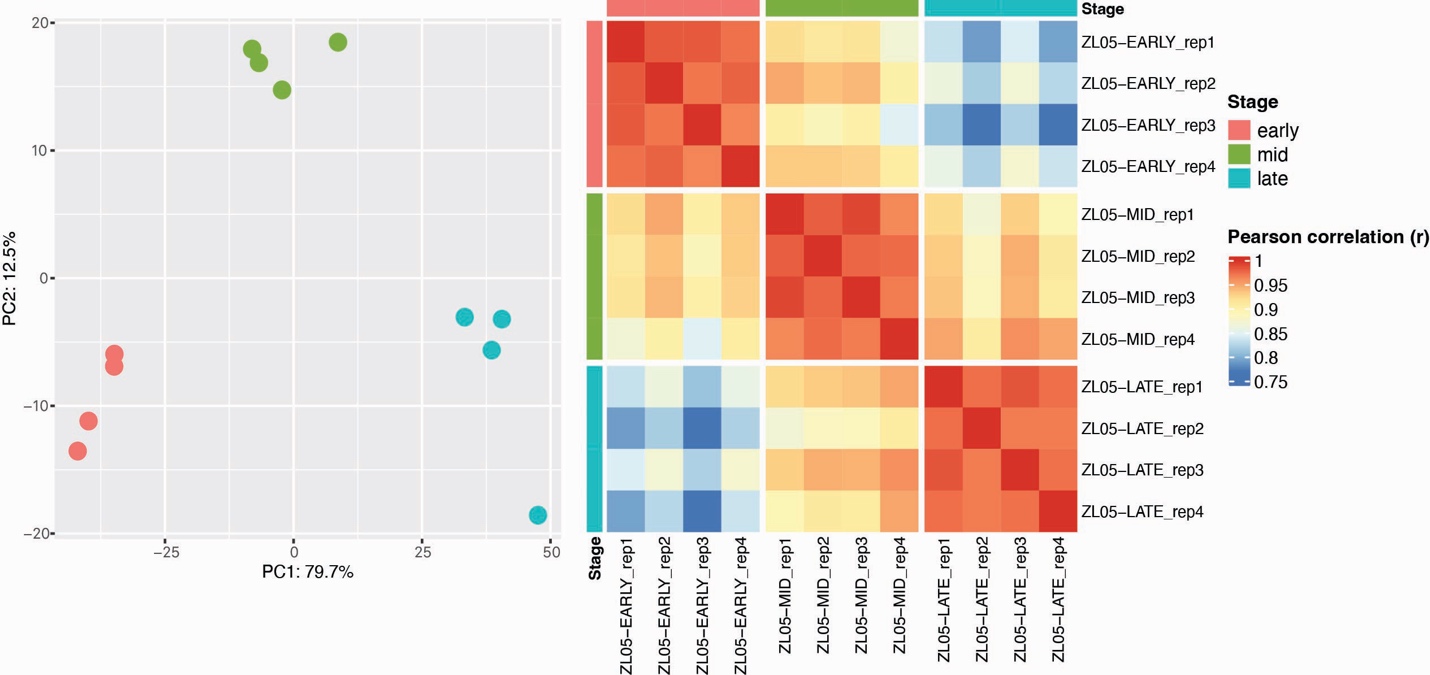


A

B

**Supplementary Figure 3.** Quality control of the replicates used for RNA-seq analysis. **(A)** PCA plot of the RNA-seq expression of the 12 abscission zone samples across early, middle, and late development. **(B)** Sample-sample Pearson correlation of variance-stabilized counts.


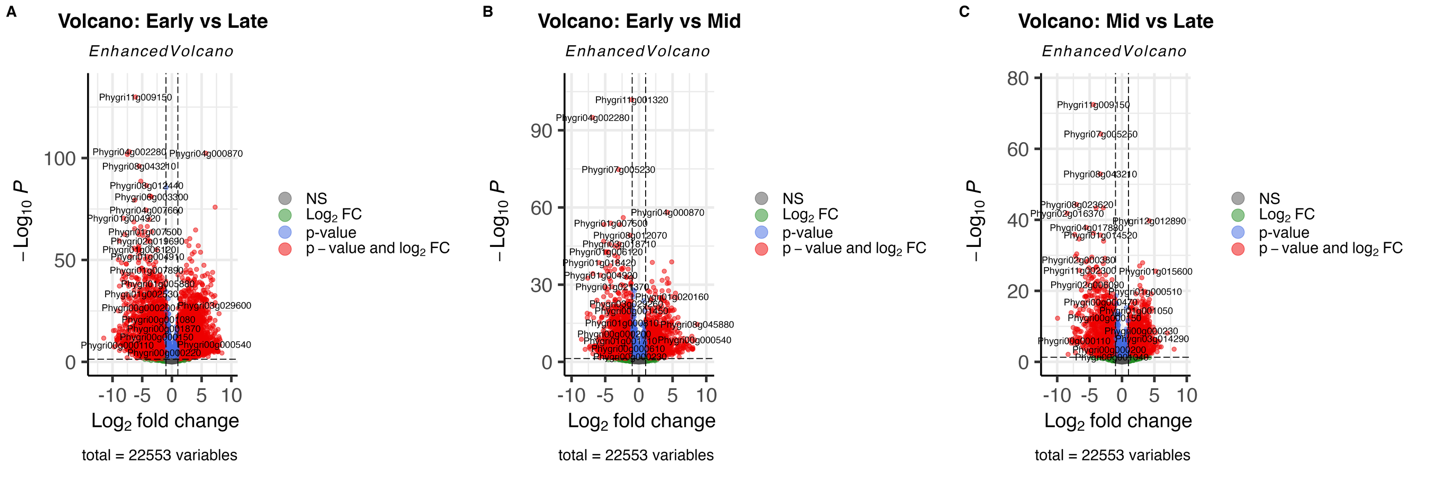


**Supplementary Figure 4.** Volcano plots of each stage comparison of the full data set (22,553 genes). Dot color representation: gray, non-significant genes; green, genes with a log2 fold change less than -1 or greater than 1; blue, genes with a significant p-value <0.05; red, genes with both a significant log2 fold change and a significant p-value. The most significant gene IDs of each comparison are labeled.


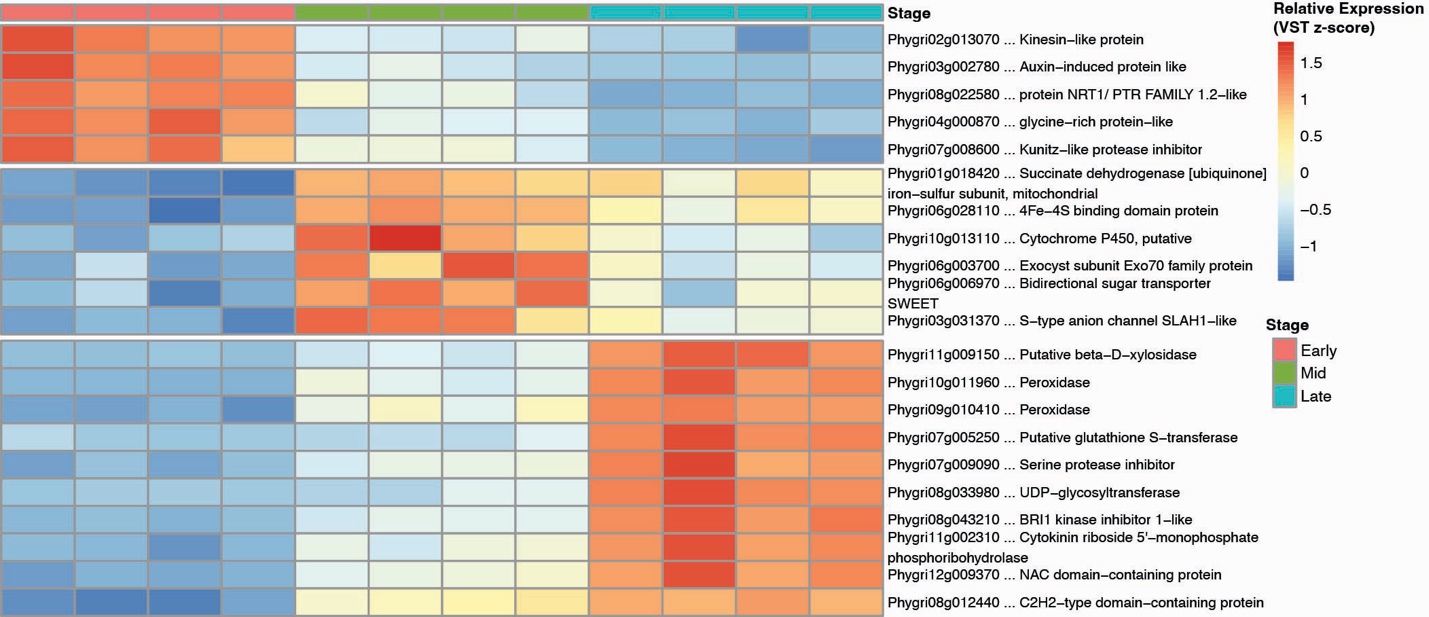


**Supplementary Figure 5.** Heatmap of representative stage-enriched genes discussed in the Results Section 3.4.5. Colors indicate the relative expression (VST z-score) of each gene where positive relative expression is red and negative expression is blue. Columns are grouped by stage where red-labeled columns are early expression levels, green are mid expression levels, and teal are late expression levels for that gene. *P*. *grisea* gene IDs and their associated descriptions are provided.


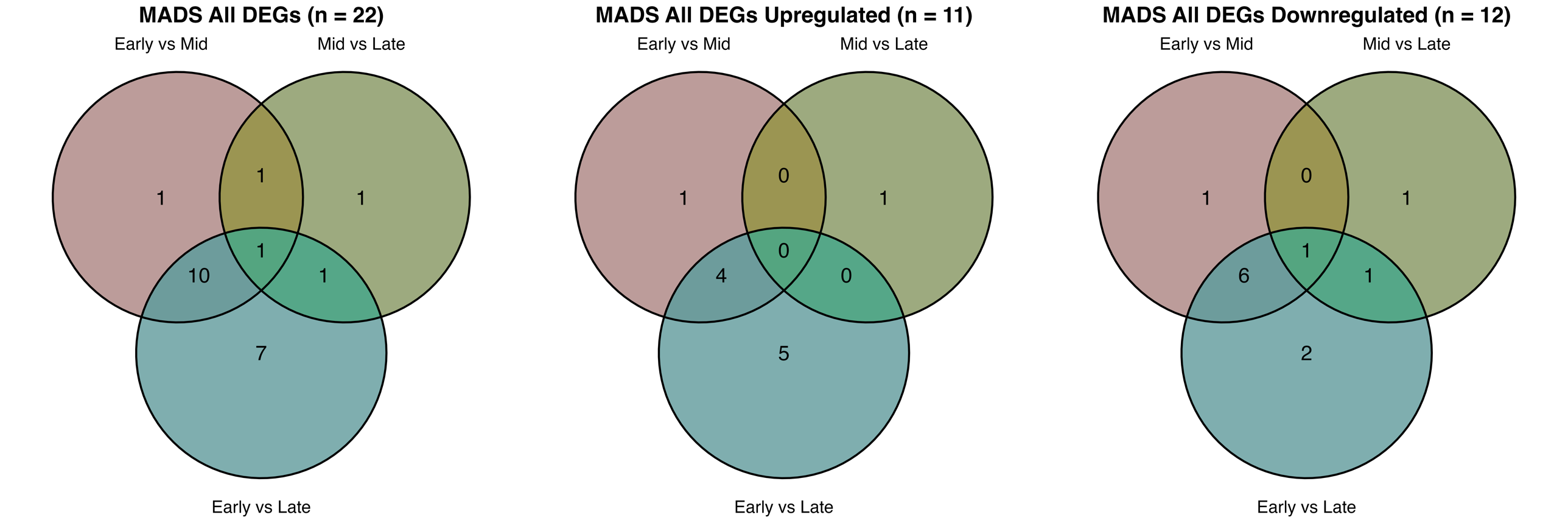


**Supplementary Figure 6.** Venn diagrams of the differentially expressed MADS-box transcription factors within the RNA-seq dataset across all comparisons between the early, mid, and late stages. **(A)** All MADS-box DEGs significant in at least one comparison regardless of direction. **(B)** Only upregulated MADS-box DEGs significant in at least one comparison. **(C)** Only downregulated MADS-box DEGs significant in at least one comparison.

**Supplementary Table 1. Holm-adjusted pairwise log-rank comparisons of treatment groups within each hormone.**

| **Hormone** | **TreatmentA** | **TreatmentB** | **chisq** | **pval** | **pval_adj** |
| --- | --- | --- | --- | --- | --- |
| **Auxin** | Auxin Distal | Auxin Proximal | 46.1350 | 1.1038E-11 | 2.2076E-11 |
| **Auxin** | Auxin Distal | Lanolin Distal | 134.0187 | 5.4123E-31 | 3.2474E-30 |
| **Auxin** | Auxin Distal | Lanolin Proximal | 131.6147 | 1.8167E-30 | 9.0833E-30 |
| **Auxin** | Auxin Proximal | Lanolin Distal | 85.4139 | 2.4201E-20 | 7.2604E-20 |
| **Auxin** | Auxin Proximal | Lanolin Proximal | 90.8043 | 1.5861E-21 | 6.3443E-21 |
| **Auxin** | Lanolin Distal | Lanolin Proximal | 0.8838 | 0.3472 | 0.34717 |
| **Ethylene** | Ethylene Distal | Ethylene Proximal | 32.7666 | 1.0392E-08 | 4.1566E-08 |
| **Ethylene** | Ethylene Distal | Water Distal | 71.8154 | 2.36301E-17 | 1.4178E-16 |
| **Ethylene** | Ethylene Distal | Water Proximal | 63.5617 | 1.5542E-15 | 7.7709E-15 |
| **Ethylene** | Ethylene Proximal | Water Distal | 18.0157 | 2.1909E-05 | 6.5727E-05 |
| **Ethylene** | Ethylene Proximal | Water Proximal | 9.7332 | 0.0018 | 0.0036 |
| **Ethylene** | Water Distal | Water Proximal | 1.7394 | 0.1872 | 0.1872 |

**Supplementary Table 2.** **Summary of quality control metrics for the 12 samples used for RNA-seq analysis.**

| **sample** | **libsize** | **detectedgenes** | **pctzeros** | **sizefactor** | **meanvst** | **medianvst** | **meanr_all** | **meanr_within** | **z_all** | **z_within** | **rz_all** | **rz_within** |
| --- | --- | --- | --- | --- | --- | --- | --- | --- | --- | --- | --- | --- |
| **ZL05-EARLY_rep1** | 13696661 | 26312 | 0.2223 | 0.9428 | 8.122 | 8.116 | 0.8918 | 0.9794 | -0.81 | 1.23 | -0.67 | 0.78 |
| **ZL05-EARLY_rep2** | 12018986 | 26153 | 0.227 | 0.8465 | 8.116 | 8.117 | 0.9117 | 0.9773 | 0.12 | 0.69 | 0 | 0.3 |
| **ZL05-EARLY_rep3** | 17702167 | 26753 | 0.2093 | 1.2129 | 8.122 | 8.128 | 0.8745 | 0.9724 | -1.61 | -0.56 | -1.25 | -0.8 |
| **ZL05-EARLY_rep4** | 15868580 | 26565 | 0.2148 | 1.1765 | 8.078 | 8.104 | 0.9087 | 0.9718 | -0.02 | -0.72 | -0.1 | -0.94 |
| **ZL05-LATE_rep1** | 10499886 | 25530 | 0.2454 | 0.6956 | 8.097 | 8.093 | 0.9119 | 0.9772 | 0.13 | 0.66 | 0 | 0.28 |
| **ZL05-LATE_rep2** | 19450248 | 26836 | 0.2068 | 1.3143 | 8.072 | 8.061 | 0.8766 | 0.9691 | -1.51 | -1.41 | -1.18 | -1.54 |
| **ZL05-LATE_rep3** | 14943675 | 26204 | 0.2255 | 1.0468 | 8.069 | 8.081 | 0.9187 | 0.9747 | 0.44 | 0.03 | 0.23 | -0.28 |
| **ZL05-LATE_rep4** | 10158448 | 25473 | 0.2471 | 0.7263 | 8.044 | 8.078 | 0.8895 | 0.971 | -0.91 | -0.93 | -0.75 | -1.13 |
| **ZL05-MID_rep1** | 13467764 | 26013 | 0.2311 | 0.9076 | 8.105 | 8.093 | 0.932 | 0.9781 | 1.06 | 0.88 | 0.68 | 0.47 |
| **ZL05-MID_rep2** | 16326389 | 26615 | 0.2133 | 1.1606 | 8.089 | 8.102 | 0.9331 | 0.9775 | 1.11 | 0.73 | 0.71 | 0.34 |
| **ZL05-MID_rep3** | 21362465 | 26621 | 0.2132 | 1.4434 | 8.107 | 8.101 | 0.9351 | 0.9785 | 1.2 | 0.99 | 0.78 | 0.57 |
| **ZL05-MID_rep4** | 12229212 | 26205 | 0.2255 | 0.8655 | 8.073 | 8.084 | 0.9261 | 0.9684 | 0.79 | -1.6 | 0.48 | -1.71 |

**Supplementary Table 3.** **Gene-gene Pearson’s correlation of *Physalis grisea* MADS-box transcription factors to *PgJ*.**

| **Gene ID** | **r** | **Tomato Ortholog** |
| --- | --- | --- |
| Phygri08g035370 | 1 | *J* |
| Phygri08g005970 | 0.999999 | *SlMADS51* |
| Phygri02g025310 | 0.999651 | *SlMBP10* |
| Phygri08g018110 | 0.989686 | *SlMBP6/SlAGL6* |
| Phygri07g015380 | 0.986551 | *SlMADS98/SlCMB1* |
| Phygri12g018350 | 0.941825 | *MC* |
| Phygri07g009060 | 0.933056 | *TM8/TDR8* |
